# System for Stable β-Estradiol-Inducible Gene Expression in the Moss *Physcomitrella* *patens*

**DOI:** 10.1371/journal.pone.0077356

**Published:** 2013-09-27

**Authors:** Minoru Kubo, Akihiro Imai, Tomoaki Nishiyama, Masaki Ishikawa, Yoshikatsu Sato, Tetsuya Kurata, Yuji Hiwatashi, Ralf Reski, Mitsuyasu Hasebe

**Affiliations:** 1 National Institute for Basic Biology, Okazaki, Japan; 2 ERATO, Japan Science and Technology Agency, Okazaki, Japan; 3 Plant Biotechnology, Faculty of Biology, University of Freiburg, Freiburg, Germany; 4 FRIAS – Freiburg Institute for Advanced Studies, Freiburg, Germany; 5 Department of Biology, Duke University, Durham, North Carolina, United States of America; 6 Advanced Science Research Center, Kanazawa University, Kanazawa, Japan; 7 School of Life Science, The Graduate University for Advanced Studies, Okazaki, Japan; 8 BIOSS – Centre for Biological Signalling Studies, Freiburg, Germany; University of Massachusetts Amherst, United States of America

## Abstract

Inducible transgene expression provides a useful tool to analyze gene function. The moss *Physcomitrella* *patens* is a model basal land plant with well-developed research tools, including a high efficiency of gene targeting and substantial genomics resources. However, current systems for controlled transgene expression remain limited. Here we report the development of an estrogen receptor mediated inducible gene expression system, based on the system used in flowering plants. After identifying the appropriate promoters to drive the chimeric transducer, we succeeded in inducing transcription over 1,000-fold after 24 h incubation with β-estradiol. The *P* *. patens* system was also effective for high-level long-term induction of gene expression; transcript levels of the activated gene were maintained for at least seven days on medium containing β-estradiol. We also established two potentially neutral targeting sites and a set of vectors for reproducible expression of two transgenes. This β-estradiol-dependent system will be useful to test genes individually or in combination, allowing stable, inducible transgenic expression in *P* *. patens* *.*

## Introduction

The moss *Physcomitrella* *patens* lineage diverged from the flowering plant lineage approximately 450 million years ago [1,2] and comparisons of its physiology and development to those in flowering plants are useful to elucidate generality, diversity, and evolution in land plants. Various genomics resources including the genome sequence [3,4] and full-length cDNA clones [5] as well as efficient gene targeting via homologous recombination [6] enable analysis of gene functions in *P* *. patens* [7,8]. Transgenic gain-of-function and loss-of-function experiments are useful to investigate gene function. For example, systems to induce transgene expression by addition of external triggers are effective tools to investigate gene functions at specific stages [9–11]. Moreover, the availability of inducible and continuously strong promoters would facilitate the production of complex biopharmaceuticals in the moss bioreactor [12]. In *P* *. patens* , a heat shock promoter derived from the upstream sequence of *Gmhsp17.3B* can be used to conditionally induce transgene expression in a short time and in all examined tissues [13,14]. However, continuous induction is difficult because long exposure to heat stress results in eventual attenuation of promoter activity [15] and seriously affects growth. The tetracycline repression system can also be used as an inducible expression system in *P* *. patens* [16], but maintaining repression of transgene expression requires cultivating the moss on medium supplemented with tetracycline, which causes growth retardation [13]. Another chemically inducible expression system is the GVG system [13,17], which uses the DNA binding domain of the yeast GAL4 transcription factor, the transcriptional activation domain of the herpes viral protein VP16, and the mammalian glucocorticoid receptor. Activation of GVG with dexamethazone elicits expression of a set of defense related genes and growth retardation in flowering plants [18–20], although its effects on *P* *. patens* have not been well examined. Based on mammalian expression technologies several inducible and autoregulated promoters have been introduced into *P* *. patens* protoplasts [21] but have not been examined yet in stably transformed moss lines during the life cycle.

To establish a more feasible gene induction system in *P* *. patens* , we employed the XVE chimeric transcription activator, which has been effectively used in flowering plants and is composed of the DNA-binding domain of the bacterial repressor LexA, the transcriptional activation domain VP16, and the carboxyl region of the human estrogen receptor [22]. When activated by exogenous β-estradiol, XVE protein binds to the LexA operator and recruits RNA polymerase II to the cauliflower mosaic virus (CaMV) minimal 35S promoter (Pm35S) [23] to induce expression of the downstream gene. This system has no apparent toxic physiological effects in *A. thaliana* [22]. To adapt this system for *P* *. patens* , we screened for promoters to drive *XVE* expression at an appropriate level for transactivation of the target gene in protonema and gametophore cells. We also examined induction and duration of transactivation of genes in *P* *. patens* .

Genetic and epigenetic effects of the insertion site and surrounding regions affect transgene expression, which can thus vary among transgenic lines even if the same construct is transformed [24,25]. To avoid this problem, we established gene targeting sites that are located at putative neutral genomic positions. Furthermore, establishment of more than one site enabled simultaneous induction of two transactivated genes. This system has been successfully used in recent publications without detailed description [26,27] and here we fully describe the useful gene induction system in *P* *. patens* .

## Results

### Screening of promoters to induce the XVE gene in *P* *. patens*

**Figure 1:**
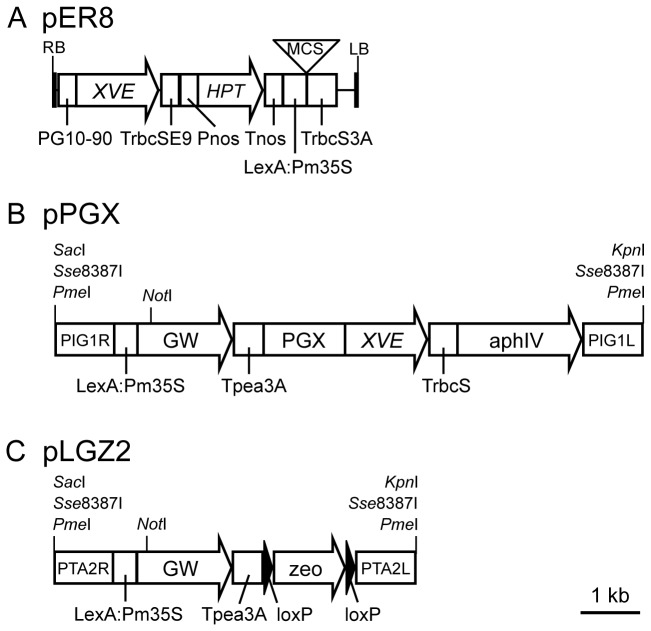
Schematic representation of the XVE system. Schematic representations of pER8 (A: modified from [22]), pPGX (B), pLGZ2 (C) vectors. RB: right border [22], PG10-90: a G10-90 synthetic promoter [32], TrbcSE9: a Pea rbcS E9 terminator [33], Pnos: Agrobacterium nopaline synthase promoter [17], *HPT*: the hygromycin phosphotransferase gene [17], Tnos, an agrobacterium nopaline synthase terminator [17], LexA:Pm35S: eight copies of LexA operators [22] connected to CaMV minimal 35S promoter [23], MCS: multi cloning site including *Xho*I, *Asc*I, *Apa*I, *Pac*I, and *Spe*I. TrbcS: a TrbcS terminator [34], LB: left border [22], PIG1R and PIG1L: DNA fragments for homologous recombination to the PIG1 putative neutral site [14], GW: the Gateway cassette rfA containing the *chloramphenicol* *resistance* gene and the *ccdB* gene flanked by attR1 and attR2 sites (Life Technologies), Tpea3A: a Tpea3A terminator [34], PGX: one of GX promoters, *XVE*: the chimeric gene with a LexA-binding [28,29], a VP16 activator [30], and an estrogen receptor domain [31], aphIV: the hygromicin phosphotransferase expression cassette [35,36], PTA2R and PTA2L: DNA fragments targeting to the PTA2 putative neutral site, loxP: the sequences for site-specific recombination by Cre recombinase [55], zeo: the bleomycin resistance protein expression cassette [36]. Backbone of pER8 is pZP200 [22]. Backbones of pPGX and pLGZ2 are pBluescriptII [49].

We modified the XVE construct, pER8 ( [22]: Figure 1A) containing the following three regions: (1) the *XVE* chimeric gene encoding a DNA-binding domain of the bacterial repressor LexA (X [28,29]:), the transcriptional activation domain of VP16 (V [30]:), and the regulatory region of the human estrogen receptor (E [31]:), which is driven by the synthetic G10-90 promoter [32] and connected to *Pisum sativum rbcS* E9 terminator [33], (2) the *HYGROMYCIN PHOSPHOTRANSFERASE* (*HPT* [17]:) gene cassette composed of a nopaline synthase promoter (Pnos [17]:) and a nopaline synthase terminator (Tnos [17]:) for selection of transformants, and (3) eight copies of a bacterial LexA operator [22] connected to Pm35S [23], a multiple cloning site to introduce a transgene, and a *Pisum sativum* pea 3A terminator [34]. For use in the moss *Physcomitrella* *patens* , we modified pER8 as follows (Figure 1B). First, we replaced the selection cassette with a marker that is effective in *P* *. patens* . The *HPT* cassette was replaced with the aphIV cassette, which uses a modified CaMV35S promoter and CaMV35S terminator that are efficient in *P* *. patens* [35,36]. Second, to facilitate cloning, the multiple cloning site was replaced by the Gateway cassette (Life Technologies). Third, to prevent position effects of insertion sites and to obtain stable expression, we used the *P* *. patens* inter-genic 1 (PIG1) site as a potential neutral site for site-specific insertion. In PIG1, no putative transcripts, loci, or repeat sequences have been assigned and no effects of gene insertion have been observed [14]. When we introduced the modified construct to *P* *. patens* , we could not detect induction of a transgene. Seeing that CaMV 35S promoter activity is different between seed plants and *P* *. patens* [37], we suspected the G10-90 promoter activity in *P* *. patens* and replaced the promoter used to express *XVE* with a promoter that works in *P* *. patens* . The G10-90 promoter induces the *XVE* gene in *A. thaliana* and tobacco [22] but we could not detect induction in *P* *. patens* protonemata. To find appropriate promoters, we searched previously published transcriptome data [38] for genes constitutively expressed in both protonemata and gametophores at similar levels and selected four promoter regions of genes that showed different expression levels. The two genes encode an RNA-binding domain protein (XM_001758956) and a kinesin protein KINID1a (AB434497 [36]:), whose promoter and 5’ untranslated regions 976 and 1517 bp upstream from their start codons were designated as GX6 and GX8, respectively. The G10-90 promoter driving *XVE* was replaced by each of these promoter fragments. The resulting constructs were designated pPGX6 (AB537481) and pPGX8 (AB537482).

**Figure 2:**
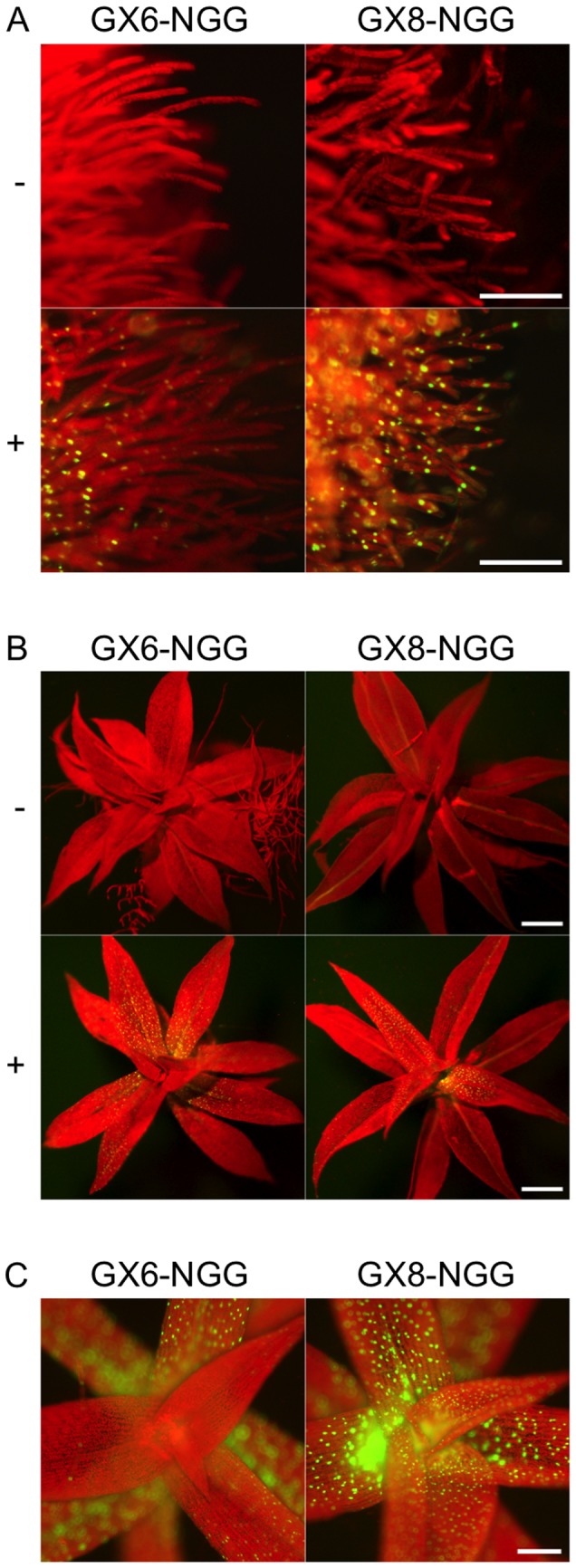
Spatial expression patterns of NGG induced by *P* *. patens* XVE system. Fluorescence images of protonemata (A) and gametophores (B) of GX6-NGG#63 and GX8-NGG#4 lines. Fluorescent signals were observed after 24 h inoculation in water with (+) or without (-) 1 µM β-estradiol. Yellowish color of leaf veins in GX8-NGG#4 is caused by reflection but not by NGG signals. (C) Magnified views of apexes of gametophores in GX6-NGG#63 and GX8-NGG#4 lines. Pictures in A, B, and C panels are at the same magnification. Bars = 100 µm.

To monitor the activity of each GX promoter driving *XVE*, we examined the expression of the marker *NLS-GFP-GUS* (*NGG*), a fusion gene composed of a nuclear localization signal (*NLS* [39]:), the *green fluorescent protein* (s*GFP* [40]:) gene, and the *uidA* (*GUS* [41]:). The *NGG* marker gene was introduced with the Gateway system into each of the pPGX constructs, downstream of the LexA:Pm35S promoter (Figure 1B). These constructs were used to transform *P* *. patens* protoplasts with a conventional PEG-mediated method; insertion into the PIG1 site was confirmed by DNA gel blot analyses (Figures S1 and S2). We selected three single-insertion lines for each GX-NGG construct and examined expression of NGG in each line after 24 h incubation with or without β-estradiol (Figures 2 and S3). The ideal promoter for driving *XVE* would produce no NGG signal in the absence of β-estradiol, but would produce sustained, high-level NGG signal in protonema and gametophore cells following induction. Without β-estradiol, fluorescent NGG signals were not detected in any protonema or gametophore cells of GX6-NGG and GX8-NGG lines. With β-estradiol, spatial patterns of NGG signals were not distinguishable between GX-NGG lines with the same GX promoters but differed among different GX promoters (Figures 2 and S3). In GX6-NGG lines, NGG signals were detected in most of protonema cells (Figure S3A). Most chloronema cells retained NGG signals except a part of chloronema apical cells, although we cannot detect morphological differences between chloronema apical cells with or without NGG signals. We could not detect NGG signals in caulonema cells. The first to approximately third leaf from a gametophore tip and younger leaves did not show NGG signals while the fourth and older leaves retained NGG signals. In GX8-NGG lines, NGG signals were detected in all cells in protonemata and gametophores.

**Figure 3:**
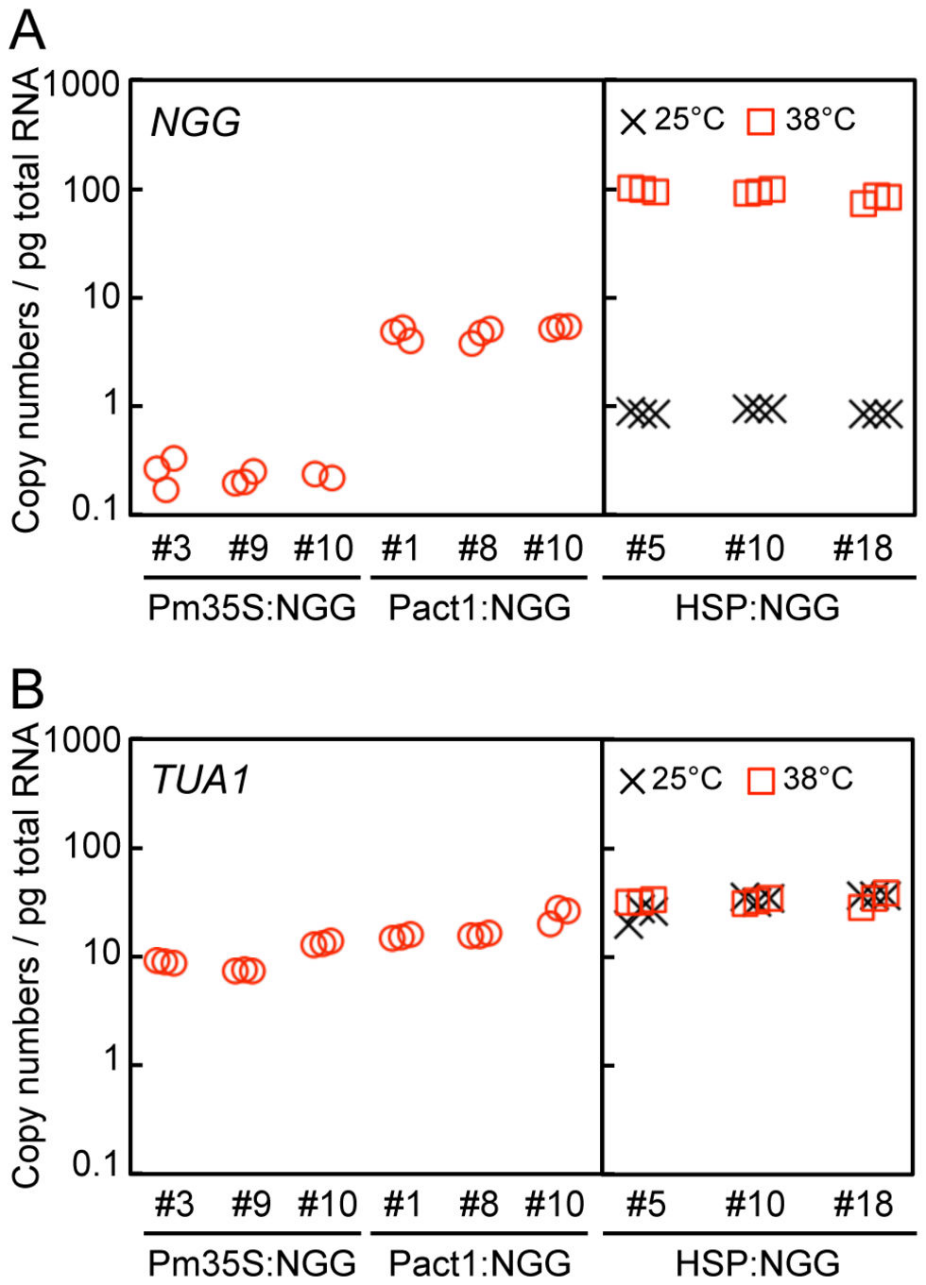
Amounts of *NGG* and *TUA1* transcripts in Pm35S:NGG, Pact1:NGG, and HSP:NGG lines. (A, B) Transcript amounts of *NGG* (A) and *TUA1* (B) in protonemata incubated for 8 days after propagation. Three independently transformed lines harboring a single copy of each DNA fragment at the PIG1 targeting site were analyzed (Figures S4 to S6). Copy numbers of the *NGG* and *TUA1* transcripts per pg total RNA were estimated by RT-qPCR. In HSP:NGG lines, protonemata were cultured for 8 days at 25°C and collected after 1 h incubation at 25°C (crosses) or 38°C (squares).

To compare the induction ability of the *P* *. patens* XVE system to other systems, each Pm35S [23] lacking the transcriptional enhancer region as a negative control, the rice *Actin1* promoter (Pact1 [16,42]:), and the soybean heat-shock protein *Gmhsp17.3B* promoter (HSP [13,14]:) were fused with *NGG* and introduced at the PIG1 site to obtain singly inserted transgenic lines (Figure S4, S5, S6). We quantified *NGG* transcripts in protonemata for each line by quantitative real time PCR (RT-qPCR). *ALPHA TUBULIN 1* (*TUA1*) was used as a control because it has been reported to be expressed in several tissues and conditions in *P* *. patens* at similar levels [26,36]. *NGG* transcripts accumulated at 0.3 ± 0.1 and 4.8 ± 0.3 copies per pg total RNA in Pm35S:NGG and Pact1:NGG lines, respectively (mean ± SD calculated based on biological replicates) (Figure 3A). By contrast, in HSP:NGG lines, *NGG* transcripts accumulated at 91.1 ± 3.0 and 0.9 ± 0.0 copies per pg total RNA with or without heat shock for 1 h at 38°C, respectively. Transcripts of *NGG* induced by heat shock attenuated to approximately one tenth of maximal levels after 24 h of incubation at 38°C (Figure S7) [13]. Transcripts of *TUA1* in Pm35S:NGG and Pact1:NGG accumulated at 8.3 ± 0.1 and 14.8 ± 0.3 copies per pg total RNA, respectively. In HSP:NGG lines, transcripts of *TUA1* were 30.1 ± 0.6 and 31.6 ± 1.2 copies per pg total RNA with and without heat shock for 1 h at 38°C, respectively (Figure 3B).

**Figure 4:**
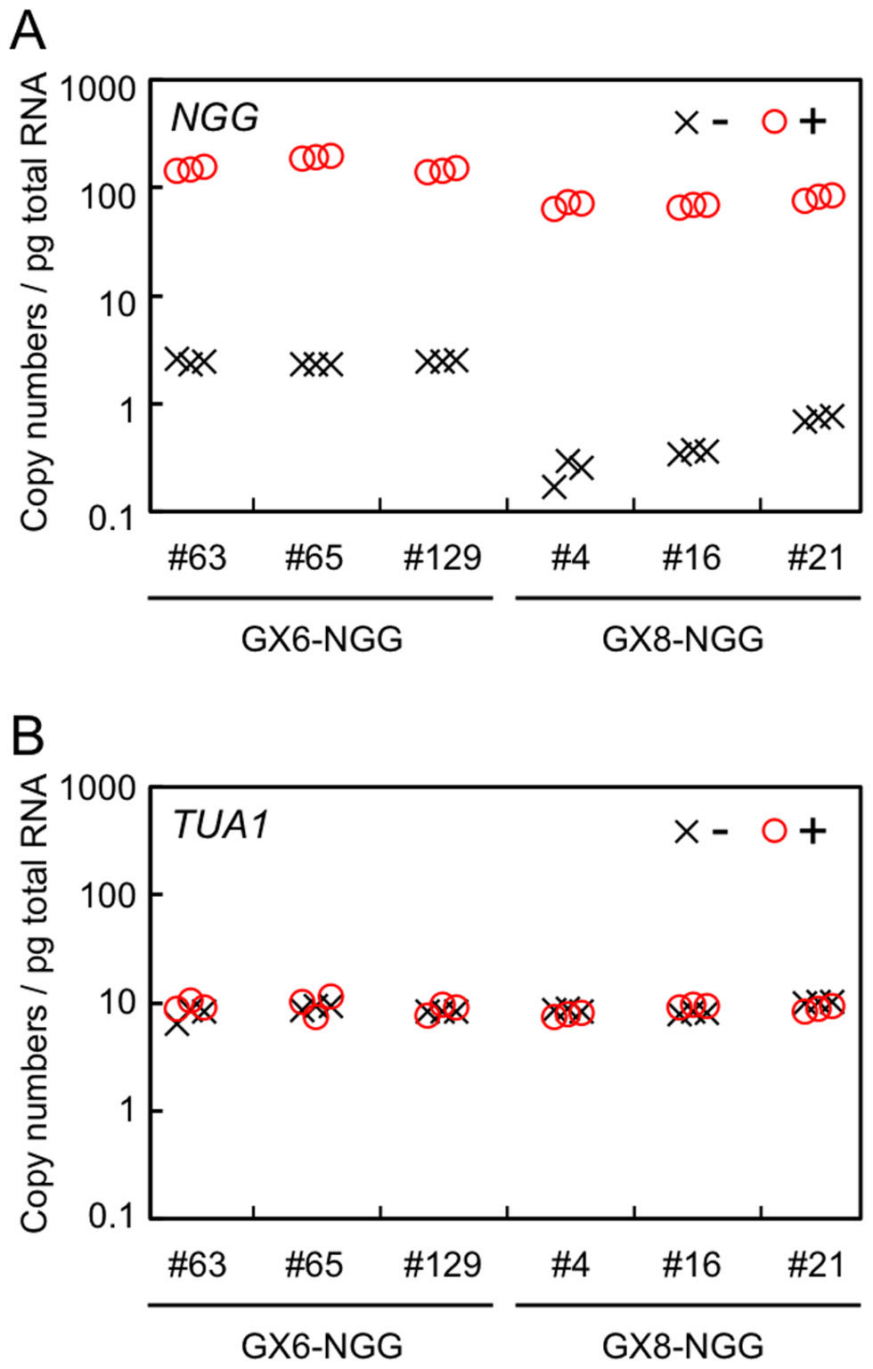
Amounts of *NGG* and *TUA1* transcripts in GX6-NGG and GX8-NGG lines. (A, B) Transcript amounts of *NGG* (A) and *TUA1* (B) in protonemata immersed in water with (+: circles) or without (-: crosses) 1 µM β-estradiol for 24 h before sampling. Three independently transformed lines harboring a single copy of each GX-NGG DNA fragment at the PIG1 targeting site were examined. Copy numbers of each *NGG* and *TUA1* gene per pg total RNA were estimated by RT-qPCR.

We next quantified transcript amounts of *NGG* and *TUA1* in protonemata incubated with β-estradiol for 24 h for each of GX-NGG line by RT-qPCR (Figure 4). Each protonema sample was incubated with or without β-estradiol for 24 h. Without β-estradiol, 3.0 ± 0.1 and 0.4 ± 0.0 copies per pg total RNA of *NGG* transcripts accumulated in GX6-NGG and GX8-NGG lines, respectively. By contrast, with β-estradiol, 180.2 ± 13.2 and 72.7 ± 2.4 copies per pg total RNA of *NGG* transcripts accumulated in GX6-NGG and GX8-NGG lines, respectively. In the case of *TUA1* transcripts, we could not detect significant differences in any line with or without β-estradiol. Independent transgenic lines harboring the same single GX-NGG construct showed similar *NGG* transcript amounts with or without β-estradiol. These indicate that relative transcript levels of *NGG* increased 54.8 ± 15.1 and 171.6 ± 80.7 times in GX6-NGG and GX8-NGG lines, respectively.

### Dose and time responsiveness to β-estradiol

**Figure 5:**
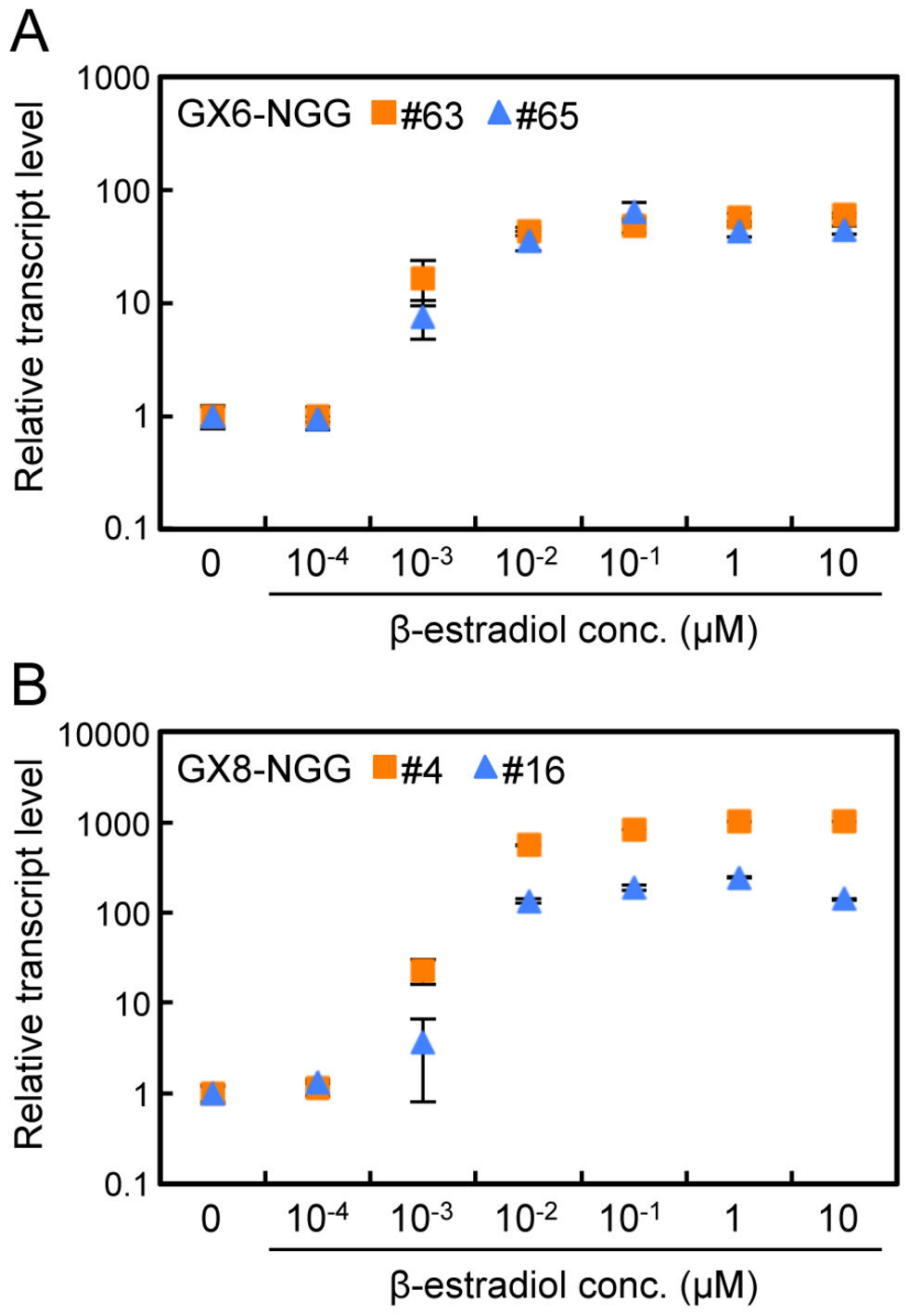
Dose responsiveness to β-estradiol in the GX6-NGG and GX8-NGG lines. (A, B) Relative transcript levels of *NGG* in protonemata of GX6-NGG#63 (squares) and GX6-NGG#65 (triangles) line (A) and GX8-NGG#4 (squares) and GX8-NGG#16 (triangles) line (B). Protonemata were immersed in water containing different concentrations of β-estradiol for 24 h before sampling. Relative transcript levels of *NGG* are normalized to *TUA1* levels and standardized to a normalized transcript level of protonemata without β-estradiol. Error bars indicate SD of the mean (n = 3).

We selected GX6 and GX8 promoters in the following experiments because they showed higher induction rates than the other two promoters. To analyze the dose responsiveness of GX6-NGG and GX8-NGG lines to β-estradiol, transcript levels of *NGG* were measured with RT-qPCR using protonemata incubated in different β-estradiol concentrations (Figure 5). *NGG* transcripts of both lines were induced at 0.001 µM and higher concentrations. Transcript levels were maximal at around 1 µM β-estradiol in all lines.

**Figure 6:**
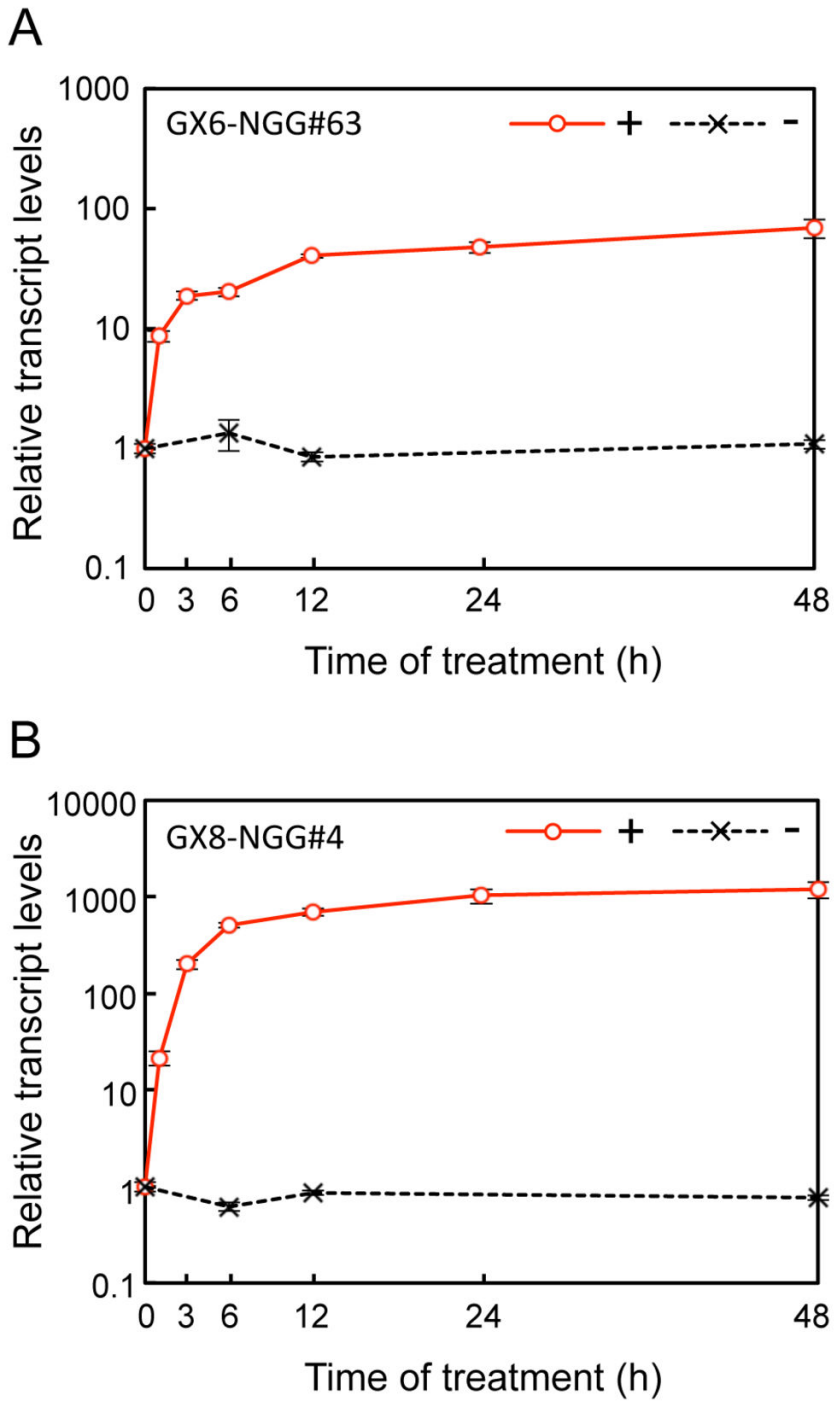
Time course responsiveness to β-estradiol in the GX6-NGG and GX8-NGG lines. (A, B) Relative transcript levels of *NGG* in protonemata of GX6-NGG#63 (A) and GX8-NGG#4 (B) lines. Relative transcript levels of *NGG* are normalized to *TUA1* and then standardized to the normalized transcript level of protonemata at 0 h. Protonemata were immersed in water with (+: circles) 1 µM β-estradiol and collected after 0, 1, 3, 6, 12, 24, and 48 h. As a negative control, protonema cultivated without (-: crosses) β-estradiol were collected after 0, 6, 12, and 48 h. Error bars indicate SD of the mean (n = 3).

The temporal change in *NGG* transcript levels was monitored in GX6-NGG#63 and GX8-NGG#4 protonemata after β-estradiol supplementation (Figure 6). *NGG* transcripts increased approximately 10- and 20-fold after 1 h with β-estradiol in GX6-NGG#63 and GX8-NGG#4 lines, respectively, and mostly plateaued until 24 h. We also monitored the maintenance of transcript levels of *NGG* in protonemata cultivated with β-estradiol. When we cultivated protonemata for 7 days in water with 1 µM β-estradiol, *NGG* transcripts were maintained for 7 days at the maximal level (Figure S8) and we did not detect visible changes in the morphology and growth of protonemata and gametophores during the induction period.

### Transactivation in multiple loci

**Figure 7:**
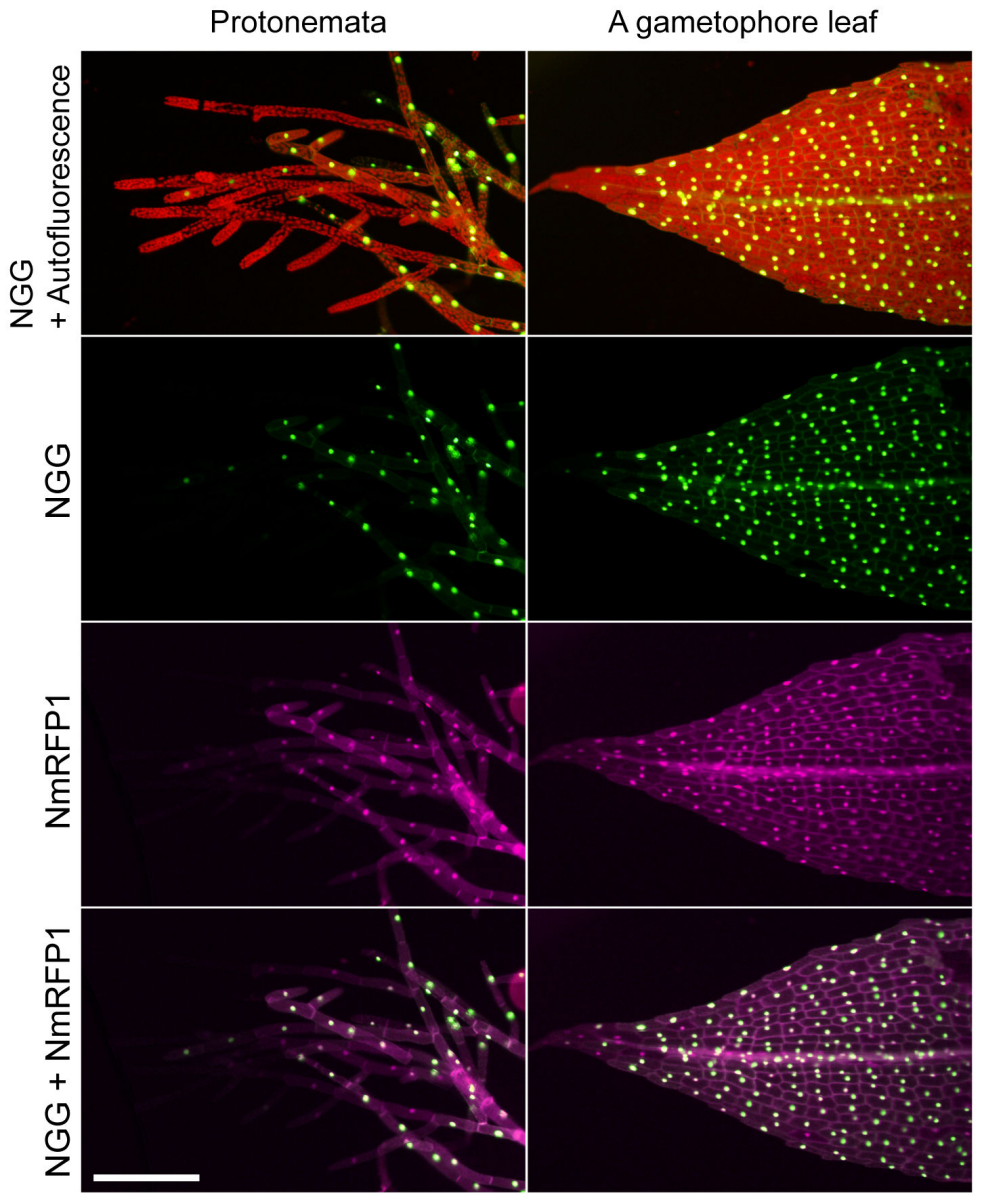
Spatial expression patterns of dually induced NGG and NmRFP1. Fluorescence images of protonemata and a gametophore leaf of the GX6-NGG#63/LGZ2-NmRFP1#11 line. They were immersed in water containing 1 µM β-estradiol for 24 h before microscopy. Fluorescence images of NGG and chlorophyll autofluorescence (top row), NGG (second row), NmRFP1 (third row), and a merged image of NGG and NmRFP1 (bottom row) are indicated. Each picture is at the same magnification. Bar = 200 µm.

**Figure 8:**
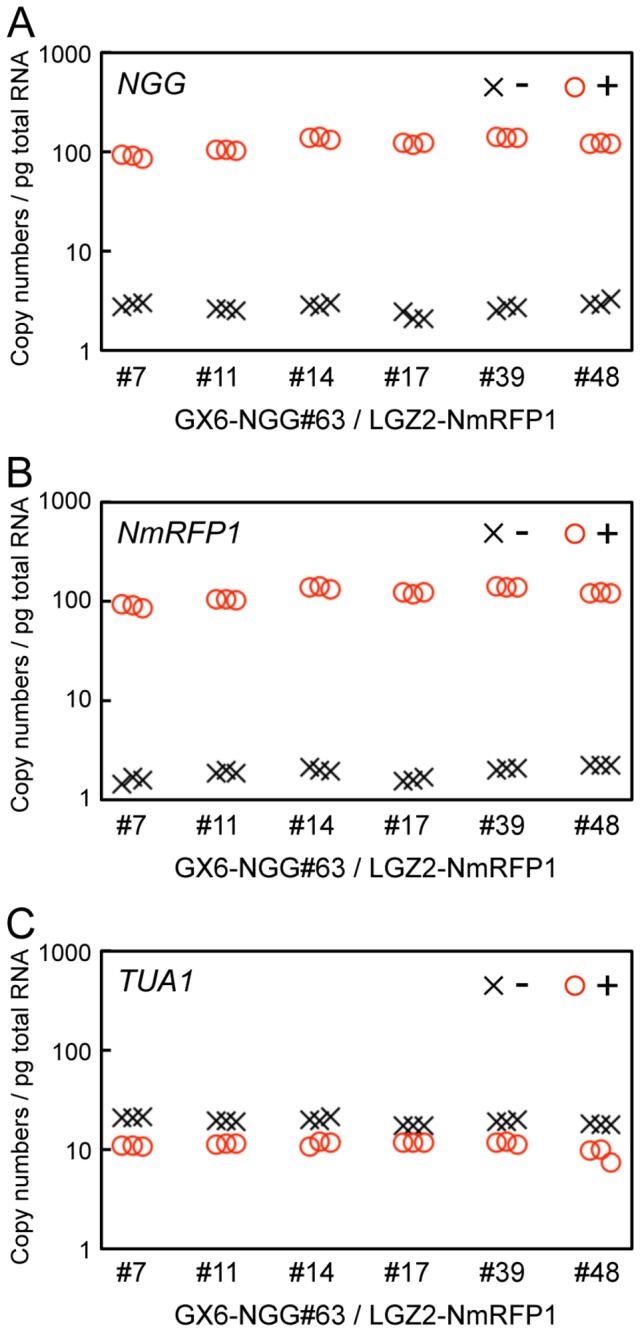
Transcript amounts for dually induced *NGG* and *NmRFP1* in transgenic *P* *. patens* lines. (A, B, C) Transcript amounts of *NGG* (A), *NmRFP1* (B), and *TUA1* (C) in protonemata of GX6-NGG#63/LGZ2-NmRFP lines. Protonemata were immersed in water with (+: circles) or without (-: crosses) 1 µM β-estradiol for 24 h before sampling. Copy numbers of *NGG*, *NmRFP1*, and *TUA1* genes per pg total RNA were estimated by RT-qPCR.

Simultaneous induction of multiple genes in a single line is useful to analyze complex biological pathways involving several genes. We examined whether the XVE system can be used to induce multiple genes introduced in the *P* *. patens* genome. In addition to the pPGX vectors (Figure 1B), we constructed another vector pLGZ2 (AB602443) containing the LexA operator and the Pm35S promoter connected to the Gateway cassette to introduce a transactivated gene and the bleomycin expression cassette [36] as a selection marker for *P* *. patens* transformants (Figure 1C). The *monomeric red fluorescent protein1* (*mRFP1* [43]:) gene was fused with a nuclear localization signal and then the *NLS-mRFP1* (*NmRFP1*) was integrated into pLGZ2 with the GATEWAY system to form LGZ2-NmRFP1 DNA fragment (Figure S9A). This fragment was introduced into the potential neutral site PTA2. This site consists of one of two sister ribosomal L31-like genes, both of which are similarly expressed in protonemata and gametophores [38]. Transgenes showed similar expression levels in gametophore and protonema of the GX6-NGG#63 line, and singly-inserted transgenic lines were selected (Figure S9B). Protonemata and gametophores of these lines were not distinguished from those of wild type, indicating the PTA2 site is neutral at least in these tissues under regular cultivated conditions. When we observed these transgenic lines, NGG and NmRFP1 signals co-localized in the nuclei in 82.8% (n = 64) protonemal cells and 91.6% (n = 237) gametophore leaf cells (Figure 7). Next, we measured the transcript amounts of *NGG* and *NmRFP1* in protonemata with RT-qPCR in six independent transgenic lines (Figure 8). Transcripts of *NGG* and *NmRFP1* accumulated at similar levels in all lines in the presence of β-estradiol.

## Discussion

### Establishment of an estrogen-inducible gene expression system in *P* *. patens*

We modified the *A. thaliana* XVE system by changing the original promoter driving *XVE* to a promoter effective in *P* *. patens* (Figure 1) and obtained reproducible amounts of transcript from a transactivated gene by β-estradiol application. Transcript levels were generally similar among independent transgenic lines for each construct (Figure 4), indicating that targeting to a specific site in the genome was effective in excluding position effects and allowed reproducible induction for each construct. Spatial patterns and transcript amounts of a transactivated gene depended on the specific GX promoter regulating *XVE* (Figures 2 and 4); therefore a specific GX promoter may be chosen depending on the desired levels of transcript and tissue specificity. For example, the transactivated gene was more highly induced in GX6-NGG lines than in GX8-NGG lines (Figure 4), although we need to be careful with the leaky expression of GX6 (Figure 4A), whose level is similar to that induced by the rice actin and endogenous tubulin promoters (Figures 3A and 4B). In GX6-NGG lines, the NGG marker protein was detected in a part of protonema cells (Figures 2 and S3). By contrast, in GX8-NGG lines, NGG was detected in all cells examined (Figures 2 and S3B). These constructs will be useful for induction of specific transgenes in a wide range of cells. Cell or tissue specific induction is a useful tool for functional analyses of genes [44] and further development of the *P* *. patens* XVE system with additional promoters will enable future analysis of cell or tissue specific gene functions.

In *P* *. patens* , the XVE system offers several advantages over current systems; specifically, it offers substantial, long-term induction of gene expression in the absence of detrimental side effects. The induction of *NGG* in GX6-NGG and GX8-NGG lines was more than ten times higher than that of Pact1 [16,42] and was similar to that of HSP [13,14] (Figures 3 and 4). Transactivated gene expression was induced by 0.001 µM β-estradiol and mostly saturated around 1 µM (Figure 5). Therefore we can regulate induction levels with β-estradiol concentrations between 0.001 µM and 1 µM. The *P* *. patens* XVE system also has a longer duration of maximal gene expression, at least 7 days (Figure S8), without detectable morphological or growth defects. For the heat-shock inducible system, transcript amounts peaked at 1 h after heat shock, but started to decrease at 2 h (Figure S7). Therefore the *P* *. patens* XVE system is useful for experiments that need long-term continuous induction like the production of biopharmaceuticals in moss bioreactors [45]. However, the XVE system needs 24 h for full induction (Figure 6), but the heat shock inducible system needs 1 h for maximum induction, indicating that the heat shock system is more suitable for experiments requiring immediate induction.

### Dual gene induction with the *P* *. patens* XVE system

We successfully induced two genes in each cell when GX6-NGG and LGZ2-NmRFP1 were introduced to independent targeting loci in a single line (Figures 1 and S9). When LGZ2-NmRFP1 was introduced into transgenic lines with GX6-NGG we had to be careful as LGZ2-NmRFP1 may be targeted by homologous recombination to the sequences shared with GX6-NGG. In our experiments, we obtained appropriate integration in 16.3% of transformants (n = 49). We could not find differences in frequency to other gene targeting experiments with single copy insertion at a targeting site [46]. Therefore, dual gene induction is feasible with this system.

It is noteworthy that GX-NGG and LGZ2-NmRFP1 DNA fragments integrated in different targeting sites were similarly regulated by XVE proteins and *NGG* and *NmRFP1* transcripts were induced in similar amounts (Figure 8, Student’s t-test: p=0.3014). In addition, both proteins showed similar spatial expression patterns (Figure 7). By contrast, we compared relative transcript levels of *NGG* in GX6-NGG#63 lines to those in GX6-NGG#63/LGZ2-NmRFP1 lines with β-estradiol (Figures 4 and 8), the former was significantly higher than the latter (nonparametric ANOVA: p=0.0351, *<0.05). This difference appears to be caused by the number of targeted loci by XVE; GX6-NGG#63 lines harboring one targeted locus (*NGG*), on the other hand, GX6-NGG#63/LGZ2-NmRFP1 lines harboring two targeted loci (*NGG* and *NmRFP1*). This system will be useful to induce genes that encode proteins that function as heterodimers or as sequentially reacting enzymes. Furthermore, when a transgene is introduced to the PTA2 site of a GX6-NGG or GX8-NGG line, the NGG signal marks the cells in which the transgene should be induced. Moreover, this XVE system with dual gene induction is also applicable for loss-of-function analyses by inducing artificial microRNA [47] or inverted repeat dsRNAs with stem-loop structures [27].

### Comparison of XVE systems between *P* *. patens* and *A. thaliana*

In both *A. thaliana* and *P* *. patens* XVE systems, the transactivated gene was induced 1 h after β-estradiol application, and the transcript levels reached a maximum around 24 h [22] (Figure 6). This suggests that the activation of XVE proteins by β-estradiol proceeds with similar kinetics in both plants. By contrast, the induced transcript amount in *A. thaliana* was attenuated at 48 h after β-estradiol addition [22], but transcript levels in *P* *. patens* were maintained for at least 7 days on medium containing β-estradiol (Figure S8). Differences were also observed in dose-responsiveness to β-estradiol. Induction of the transactivated gene in *P* *. patens* (Figure 5) and *A. thaliana* [22] was detected with 0.001 µM and 0.008 µM β-estradiol, respectively, and reached a maximum at around 1 µM and 5 µM, respectively. Although the β-estradiol treatment regimen differs for these plants, these differences may also be related to diverged metabolic pathways of steroid biosynthesis and degradation between *A. thaliana* and *P* *. patens* [48].

## Materials and Methods

### Plant materials and growth conditions

The moss *Physcomitrella* *patens* Gransden 2004 strain [3] was used as a wild type and cultured on BCDAT agar medium under continuous white light at 25°C [5].

### Plasmid construction

To construct the Pm35S DNA fragment, we annealed the oligonucleotides 5’- GACCCTTCCTCTATATAAGGAAGTTCATTTCATTTGGAGAGGACACGCTGAAGCTAGTC-3’ and 5’- GACTAGCTTCAGCGTGTCCTCTCCAAATGAAATGAACTTCCTTATATAGAGGAAGGGTC-3’. To construct the Pact1 DNA fragment, the *OsACT1* promoter region [16,42] was PCR-amplified from the pTKM1 plasmid [16,42] with primers 5’- CTCAAGCTTCGAGGTCATTCATATGCTTGAG-3’ and 5’- ATCTTCTACCTACAAAAAAGCTCCG-3’. To construct the HSP DNA fragment, the *Gmhsp17.3B* promoter region was PCR-amplified from pPTA2-HSP with primers 5’- TCTAGATAGTCAGCCTTTTAAGAGATAG-3’ and 5’-P4345 HSP-blunt-r-3’. Pm35S, Pact1, and HSP DNA fragments were inserted to the pPIG1b-NGGII plasmid (AB537478) [26,36] at the *Sma*I site to construct Pm35S:NGG, Pact1:NGG, and HSP:NGG plasmids, respectively. Direction and sequences of the inserts in the constructs were confirmed by PCR and sequencing.

To construct the pPGX vectors, DNA fragments including the LexA:Pm35S and the Tpea3A terminator were PCR-amplified from the pER8 plasmid [22] with primers 5’- ATCGATAGCTTGGGCTGCAGGTCGAGGCTAAAAAAC-3’ and 5’- CGGGATCCTACGTAAAGCCTATACTGTACTTAACTTG-3’. This fragment was inserted into the pPIG1b plasmid [14] between blunted-*Xho*I and *Bam*HI sites. The *XVE* gene fragment was PCR-amplified from pER8 with primers 5’- ATGAAAGCGTTAACGGCCAGGCAACAAGAG-3’ and 5’- CTGTCGAGGGGGGATCAATTCCCCGATCTAG-3’, and it was inserted between the *Bam*HI and *Xba*I sites after blunting. The DNA fragment including LexA:Pm35S, Tpea3 terminator, and the *XVE* gene was PCR-amplified with primers 5’-ATCGATAGCTTGGGCTGCAGGTCGAGGCTAAAAAAC-3’ and 5’-TCAGACTGTGGCAGGGAAAC-3’, and inserted between blunted *Xba*I and *Stu*I sites of the pPIG1b-HSP-aphIV vector harboring PIG1bR and PIG1bL for neutral site targeting and the aphIV cassette for hygromicin resistance in *P* *. patens* . The Gateway cassette rfA (Invitrogen) was inserted into the resulting vector between the *Asc*I and *Spe*I sites to construct the pPIG1b-LGXH plasmid (AB602444).

To construct pPGX6 and pPGX8, the promoter regions were PCR-amplified from *P* *. patens* genomic DNA with the primers described in Table S1. Each GX promoter DNA fragment was inserted into pPIG1b-LGXH at the *Sna*BI site. Direction and sequences of the inserts in all constructs were confirmed by PCR and sequencing. The *NGG* gene cloned into pENTR/D/TOPO [26] was introduced into each pPGX vector by LR reaction of the GATEWAY system (Life Technologies) according to the manufacturer’s instructions. These constructs without a backbone vector are represented in Figures S1 and S2.

To select potential neutral sites, we searched for redundant loci with similar expression levels in protonemata, gametophores, and excised leaves [38]. One of these regions was designated as the *P* *. patens* targeting site 2 (PTA2), which includes parts of the *ribosomal protein L31e* gene and is located on the scaffold_58: 518177–519180 and scaffold_58: 519609-520542 (http://genome.jgi-psf.org/Phypa11/Phypa11.home.html). These regions (PTA2-3’ side and PTA2-5’ side) were PCR-amplified from *P* *. patens* genomic DNA with primers 5’-TTGTTCAGGATAATGGTTCACAAAA-3’ and 5’-GTTCTTTCTGTCATTAACTGGTTGC-3’, 5’-gtatacGCGACTAGTGAGAGAATGTTCCAG-3’ and 5’-GGGGATTAATTATTGGAGGAAAACT-3’, respectively. To construct the pPTA2r plasmid, these DNA fragments were inserted at *Stu*I and at *Eco*RV, respectively, in pBluescriptII [49] containing a multi-cloning site with *Sse*8387I, *Pme*I, *Stu*I *Eco*RV, *Sma*I, *Sse*8387I, and *Pme*I. We inserted a DNA linker with *Cla*I, *Stu*I, *Nde*I, and *Sph*I sites at *Bst*Z17I into pPTA2r, to form the pPTA2r-linker plasmid. To construct the pLGZ2 plasmid, the DNA fragment including the bleomycin resistant gene expression cassette with loxP sites, was excised between *Nde*I and *Hin*dIII from the p35S-loxP-Zeo plasmid (AB540628) and inserted between *Nde*I and *Hind*III of the pPTA2r-linker. Then, the DNA fragments including LexA:Pm35S, the Gateway cassette, and the Tpea3A terminator were PCR-amplified from pPIG1b-LGXH with primers 5’-ATCGATAGCTTGGGCTGCAGGTCGAGGCTAAAAAAC-3’ and 5’- CGGGATCCTACGTAAAGCCTATACTGTACTTAACTTG -3’, and inserted between *Cla*I and *Stu*I.

### Transformation

Transformation of *P* *. patens* with the polyethylene glycol method was carried out as described previously [50]. Appropriate targeting was confirmed by PCR and DNA gel blot analyses [26,36].

### β-estradiol treatment

β-estradiol (Wako) was dissolved to 10 mM in dimethyl sulfoxide (DMSO) (Wako) and stored at -30°C. To apply β-estradiol to GX-NGG lines, the stock solution was diluted to an appropriate concentration with sterile deionized water and then, 20 ml of β-estradiol solution was poured into a 9 cm plastic dish in which GX-NGG lines were cultivated. They were incubated at 25°C under continuous light until used for microscopy and sampling for RNA extraction.

### Microscopy

Fluorescent signals were observed by a fluorescence stereo microscope (SZX16; Olympus) and an inverted fluorescence microscope (IX-70; Olympus). Images were recorded by digital camera (DP-71 and E-PL1s; Olympus). Image intensities of NGG and NmRFP1 were adjusted to appropriate levels by Photoshop CS3 (Adobe).

### RNA extraction and RT-qPCR

Total RNA from protonemata cultured on BCDAT medium covered with cellophane for 8 days was extracted by RNeasy plant mini kit (Qiagen) with DNase treatment on columns according to the manufacturer’s instructions. To synthesize cDNAs, 1 µg of total RNA was dissolved in 11 µl of nuclease-free water and 1 µl of 0.5 µg/µl oligo-(dT)_12-18_ primer (Life Technologies) and 1 µl of 10 mM dNTP mix were added. After gently mixing, it was incubated at 65°C for 5 min, and then placed on ice for at least 1 min. Reverse transcription mixture was prepared on ice as follows: 4 µl of 5xRT buffer, 1 µl of 0.1 M DTT, 1µl of 40 U/µl RNaseOUT RNase inhibitor (Life Technologies), and 1 µl of 200 U/µl SuperScriptIII reverse transcriptase (Life Technologies). 7 µl of reverse transcription mixture was added to the pretreated RNA sample, gently mixed, and incubated at 50°C for 60 min. After inactivation of reverse transcription at 70°C for 15 min, cDNA solution was diluted ten-fold with nuclease-free water as qPCR template. 1 and 2 µl of qPCR template was used for 10 and 20 µl of qPCR mixture, respectively. Primers for qPCR are listed in Table S2 and were added to qPCR mixture at 0.3 µM each. Absolute quantification by qPCR was performed by QuantiTect SYBR, Green PCR Kit (Qiagen) with ABI PRISM 7500 (Life Technologies) following the condition: 50°C for 2 min and 95°C for 10 min as pre-treatments, 95°C for 15 sec and 60°C for 1 min at 40 cycles as amplification. After amplification cycles, we carried out dissociation analyses for confirmation of target validity. Standard curves for absolute quantification were estimated by dilution series (10, 1, 0.1, 0.01, and 0.001 pg/µl) of the following plasmids: NGG; pENTR::NGG (5.2 kb), TUA1(AB096718); pphb6e07 (4.9 kb), NmRFP1; pOG1::NmRFP1 (11.3 kb), and XVE; pPGX2b (11.6 kb). With the molecular weight of the plasmids, copy numbers of transcripts were calculated [51] as follows: Weight in Daltons (g/mol) = (bp size of plasmids) (615[Da/bp]), Hence: (g/mol)/Avogadro’s number = g/molecule = copy numbers. This calculation produces copy number equivalent in double stranded DNA; however, our templates are single stranded. These experiments were carried out and evaluated with at least two sets of biological replicates and three sets of experimental replicates were performed.

### DNA Gel-Blot Analyses

Approximately 3 µg of genomic DNA was digested with restriction enzymes, run on 0.7% (w/v) SeaKemGTG agarose (Lonza), and transferred to a Hybond-N+ nylon membrane (GE Healthcare). Probe labeling, hybridization, and detection were performed using the AlkPhos Direct Labeling and Detection System with CDP-*Star* (GE Healthcare) according to the manufacturer’s instructions [26,36].

### Statistical analysis

Statistical analysis of qPCR data was performed using R [52]. Relative transcript levels of *NGG* in GX6-NGG#63 and GX6-NGG#63/LGZ2-NmRFP1 lines deviated significantly from the normal distribution (Shapiro.test [53]). Hence, data were analyzed using nonparametric ANOVA as implemented in the nparcomp package [54].

### Accession Numbers

Sequences and information of the vectors in this work have been deposited to DDBJ/GenBank/EMBL data libraries as follows: pPIG1b-LGXH (AB602444), pPGX6 (AB537481), pPGX8 (AB537482), and pLGZ2 (AB602443).

## Supporting Information

Figure S1 **DNA gel blot analyses of *P* *. patens* the GX6-NGG transgenic line.** (A) Schematic representation of a genomic locus and the construct. LexA:Pm35S: eight copies of LexA operators [22] connected to CaMV minimal 35S promoter [23], *NGG*: the *NLS-GFP-GUS* (*NGG*) fusion gene composed of a nuclear localization signal (*NLS* [39]:), the *green fluorescent protein* (s*GFP* [40]:) gene, and the *uidA* (*GUS* [41]:) gene, Tpea3A: a Tpea3A terminator [34], PGX: one of GX promoters, *XVE*: the chimeric gene with a LexA-binding [28,29], a VP16 activator [30], and an estrogen receptor domain [31], TrbcS: a TrbcS terminator [34], aphIV: the hygromicin phosphotransferase expression cassette [35,36]. Gray bars indicate probe regions for DNA gel blot analyses. (B) DNA gel blot analyses of transgenic *P* *. patens* lines GX6-NGG. Each genomic DNA was digested with *Eco*T22I. DNA gel blot analyses of GX6-NGG#63 and GX6-NGG#129 were previously reported [26].
(TIF)

Figure S2 **DNA gel blot analyses of *P* *. patens* GX8-NGG transgenic lines.** (A) Schematic representation of a genomic locus and the construct. LexA:Pm35S: eight copies of LexA operators [22] connected to CaMV minimal 35S promoter [23], *NGG*: the *NLS-GFP-GUS* (*NGG*) fusion gene composed of a nuclear localization signal (*NLS* [39]:), the *green fluorescent protein* (s*GFP* [40]:) gene, and the *uidA* (*GUS* [41]:) gene, Tpea3A: a Tpea3A terminator [34], PGX8: GX8 promoter, *XVE*: the chimeric gene with a LexA-binding [28,29], a VP16 activator [30], and an estrogen receptor domain [31], TrbcS: a TrbcS terminator [34], aphIV: the hygromicin phosphotransferase expression cassette [35,36]. Gray bars indicate probe regions for DNA gel blot analyses. (B) DNA gel blot analyses of transgenic *P* *. patens* lines GX8-NGG. Each genomic DNA was digested with *Bgl*II.
(TIF)

Figure S3 **8-day old protonemata of GX6-NGG and GX8-NGG lines and high-resolution images of chloronema and cauronema cells in GX6-NGG lines with β-estradiol.** (A,B) Fluorescence images of 8-day old protonemata of GX6-NGG#63 (A) and GX8-NGG#4 (B) lines. Small amount of protonemata was inoculated on BCDAT agar under continuous light at 25°C for 7 days. And then, these were immersed in the water with 1 µM β-estradiol for 1 day before observation. Bright field (C, E, G) and fluorescence (D, F, H) images of chloronema (C, D, E, F) and cauronema (G, H) cells of GX6-NGG#63 lines. Arrows in D and F indicate nuclei with GFP signals. Bars: B = 1 mm. D, F, and H = 200 µm.
(TIF)

Figure S4 **DNA gel blot analysis of transgenic *P* *. patens* Pm35S:NGG lines.** (A) Schematic representation of the genomic locus and the construct. Pm35S: CaMV minimal 35S promoter [23], *NGG*: the *NLS-GFP-GUS* (*NGG*) fusion gene composed of a nuclear localization signal (*NLS* [39]:), the *green fluorescent protein* (s*GFP* [40]:) gene, and the *uidA* (*GUS* [41]:) gene, Tnos: a nos terminator [26], BSD: the blasticidin S deaminase gene cassette [26]. Gray bars indicate probe regions for DNA gel blot analyses. (B) DNA gel blot analyses of Pm35S:NGG transgenic *P* *. patens* lines. Each genomic DNA was digested with *Eco*T22I.
(TIF)

Figure S5 **DNA gel blot analysis of transgenic *P* *. patens* Pact1:NGG lines.** (A) Schematic representation of a genomic locus and the construct Pact1: the rice actin1 promoter [16,42], *NGG*: the *NLS-GFP-GUS* (*NGG*) fusion gene composed of a nuclear localization signal (*NLS* [39]:), the *green fluorescent protein* (s*GFP* [40]:) gene, and the *uidA* (*GUS* [41]:) gene, Tnos: a nos terminator [26], BSD: the blasticidin S deaminase gene cassette [26]. Other abbreviations are indicated in the legend of Figure 1. Gray bars indicate probe regions for DNA gel blot analyses. (B) DNA gel blot analyses of Pact1:NGG transgenic *P* *. patens* lines. Each genomic DNA was digested with *Hin*cII.
(TIF)

Figure S6 **DNA gel blot analysis of transgenic *P* *. patens* HSP:NGG lines.** (A) Schematic representation of a genomic locus and the construct. HSP: the soybean *Gmhsp17.3B* promoter [13,14], *NGG*: the *NLS-GFP-GUS* (*NGG*) fusion gene composed of a nuclear localization signal (*NLS* [39]:), the *green fluorescent protein* (s*GFP* [40]:) gene, and the *uidA* (*GUS* [41]:) gene, Tnos: a nos terminator [26], BSD: the blasticidin S deaminase gene cassette [26]. Gray bars indicate probe regions for DNA gel blot analyses. (B) DNA gel blot analyses of HSP:NGG transgenic *P* *. patens* lines. Each genomic DNA was digested with *Eco*T22I.
(TIF)

Figure S7 **Time-course responsiveness to heat shock treatment of the HSP:NGG#5 line.** Protonemata were incubated at 25°C (crosses) and 38°C (squares) for 0, 1, 2, 6, 12, and 24 h and then immediately collected. Relative transcript levels of *NGG* are normalized to *TUA1* and then standardized to a normalized transcript level of protonemata at 0 h. Error bars indicate SD of the mean (n = 3).
(TIF)

Figure S8 **Time course responsiveness to β-estradiol in the GX6-NGG and GX8-NGG lines.** (A, B) Relative transcript levels of *NGG* in protonemata of GX6-NGG#63 (A) and GX8-NGG#4 (B) lines. Relative transcript levels of *NGG* are normalized to *TUA1* and then standardized to a normalized transcript level of protonemata at 0 h. Protonemata were immersed in water with (+: circles) or without (-: crosses) 1 µM β-estradiol and collected after 1, 2, 4, and 7 d. Error bars indicate SD of the mean (n = 3).
(TIF)

Figure S9 **DNA gel blot analysis of transgenic *P* *. patens* GX6-NGG/LGZ2-NmRFP1 lines.** (A) Schematic representation of a genomic locus and the construct. LexA:Pm35S: eight copies of LexA operators [22] connected to CaMV minimal 35S promoter [23], *NmRFP1*: the *mRFP1* [43] gene with a nuclear localization signal [39], Tpea3A: a Tpea3A terminator [34], loxP: the sequences for site-specific recombination by Cre recombinase [55], zeo: the bleomycin resistant protein expression cassette [36]. A gray bar indicates a probe region for DNA gel blot analyses. (B) DNA gel blot analyses of GX6-NGG#63/LGZ2-NmRFP1 transgenic lines. Genomic DNA was digested with *Hin*dIII.
(TIF)

Table S1 **Primers for cloning of GX promoter regions.**
(PDF)

Table S2 **Primers for qPCR.**
(PDF)
