# Supplementary figures and images for: System for Stable β-Estradiol-Inducible Gene Expression in the Moss Physcomitrella patens
Source: PLoS One. 2013 Sep 27;8(9):e77356. doi: 10.1371/journal.pone.0077356 (PMC3785464; doi:10.1371/journal.pone.0077356)

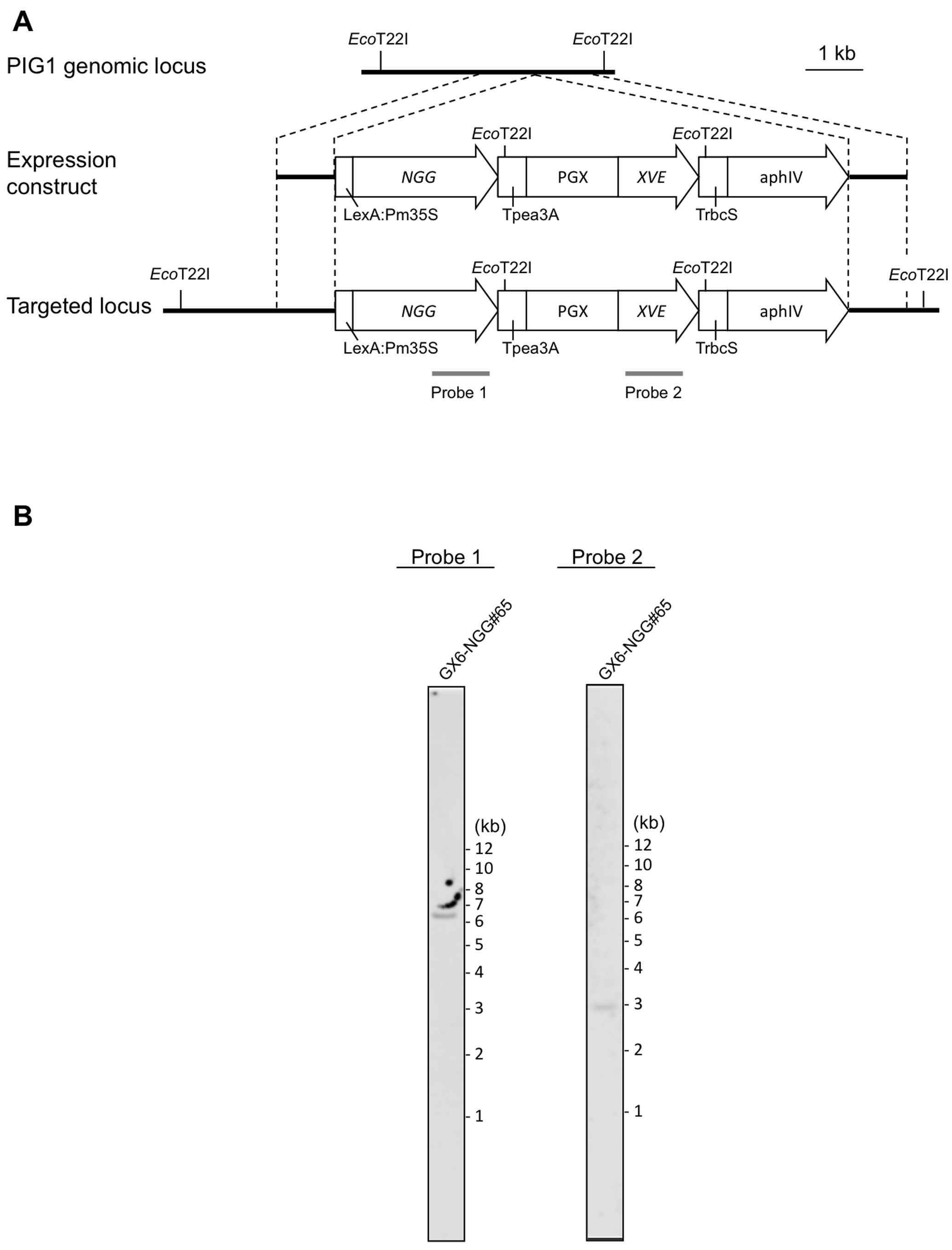

Supplement: Figure S1 — DNA gel blot analyses of P . patens the GX6-NGG transgenic line. (A) Schematic representation of a genomic locus and the construct. LexA:Pm35S: eight copies of LexA operators [22] connected to CaMV minimal 35S promoter [23], NGG: the NLS-GFP-GUS (NGG) fusion gene composed of a nuclear localization signal (NLS [39]:), the green fluorescent protein (sGFP [40]:) gene, and the uidA (GUS [41]:) gene, Tpea3A: a Tpea3A terminator [34], PGX: one of GX promoters, XVE: the chimeric gene with a LexA-binding [28,29], a VP16 activator [30], and an estrogen receptor domain [31], TrbcS: a TrbcS terminator [34], aphIV: the hygromicin phosphotransferase expression cassette [35,36]. Gray bars indicate probe regions for DNA gel blot analyses. (B) DNA gel blot analyses of transgenic P . patens lines GX6-NGG. Each genomic DNA was digested with EcoT22I. DNA gel blot analyses of GX6-NGG#63 and GX6-NGG#129 were previously reported [26]. (TIF) [file pone.0077356.s001.tif]

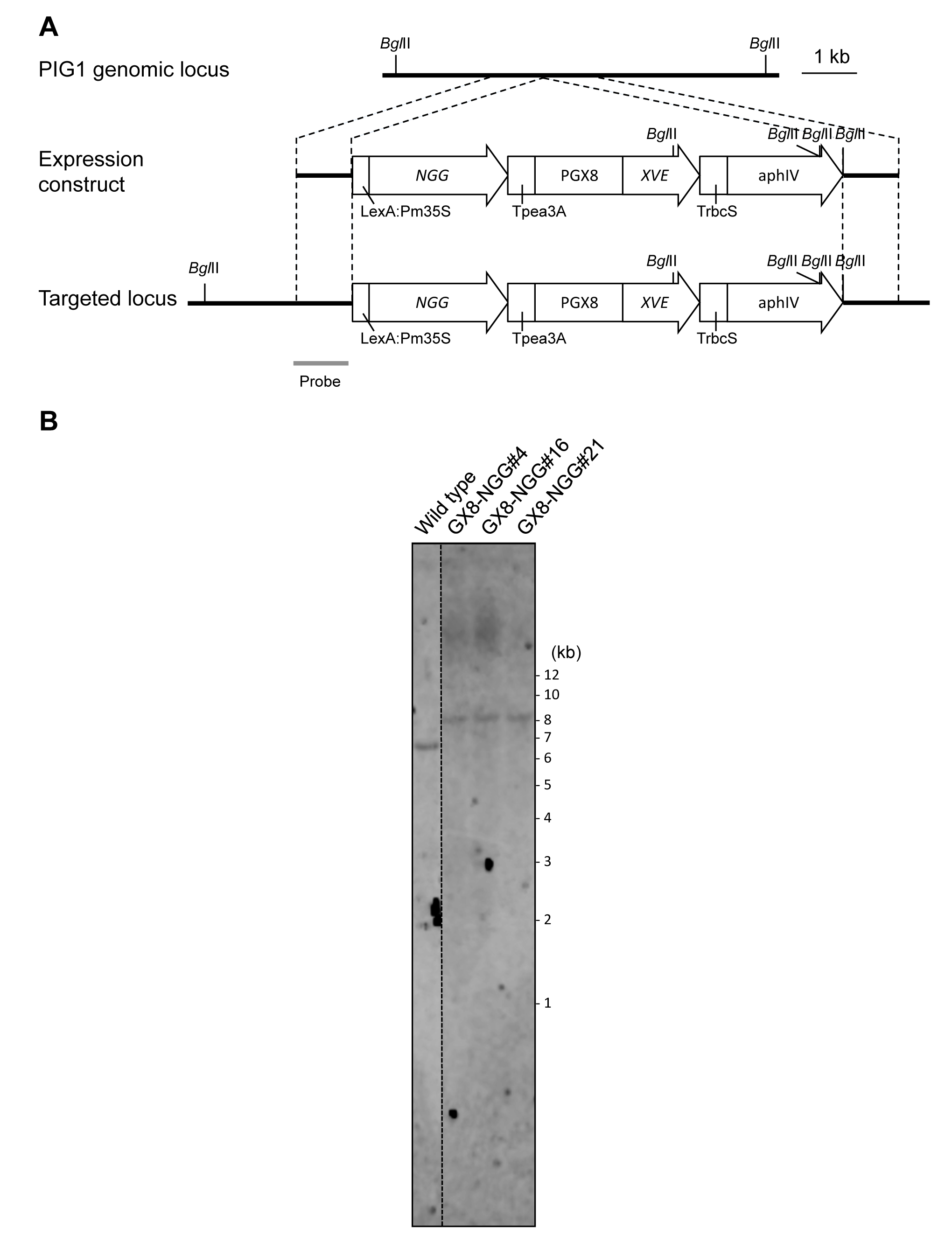

Supplement: Figure S2 — DNA gel blot analyses of P . patens GX8-NGG transgenic lines. (A) Schematic representation of a genomic locus and the construct. LexA:Pm35S: eight copies of LexA operators [22] connected to CaMV minimal 35S promoter [23], NGG: the NLS-GFP-GUS (NGG) fusion gene composed of a nuclear localization signal (NLS [39]:), the green fluorescent protein (sGFP [40]:) gene, and the uidA (GUS [41]:) gene, Tpea3A: a Tpea3A terminator [34], PGX8: GX8 promoter, XVE: the chimeric gene with a LexA-binding [28,29], a VP16 activator [30], and an estrogen receptor domain [31], TrbcS: a TrbcS terminator [34], aphIV: the hygromicin phosphotransferase expression cassette [35,36]. Gray bars indicate probe regions for DNA gel blot analyses. (B) DNA gel blot analyses of transgenic P . patens lines GX8-NGG. Each genomic DNA was digested with BglII. (TIF) [file pone.0077356.s002.tif]

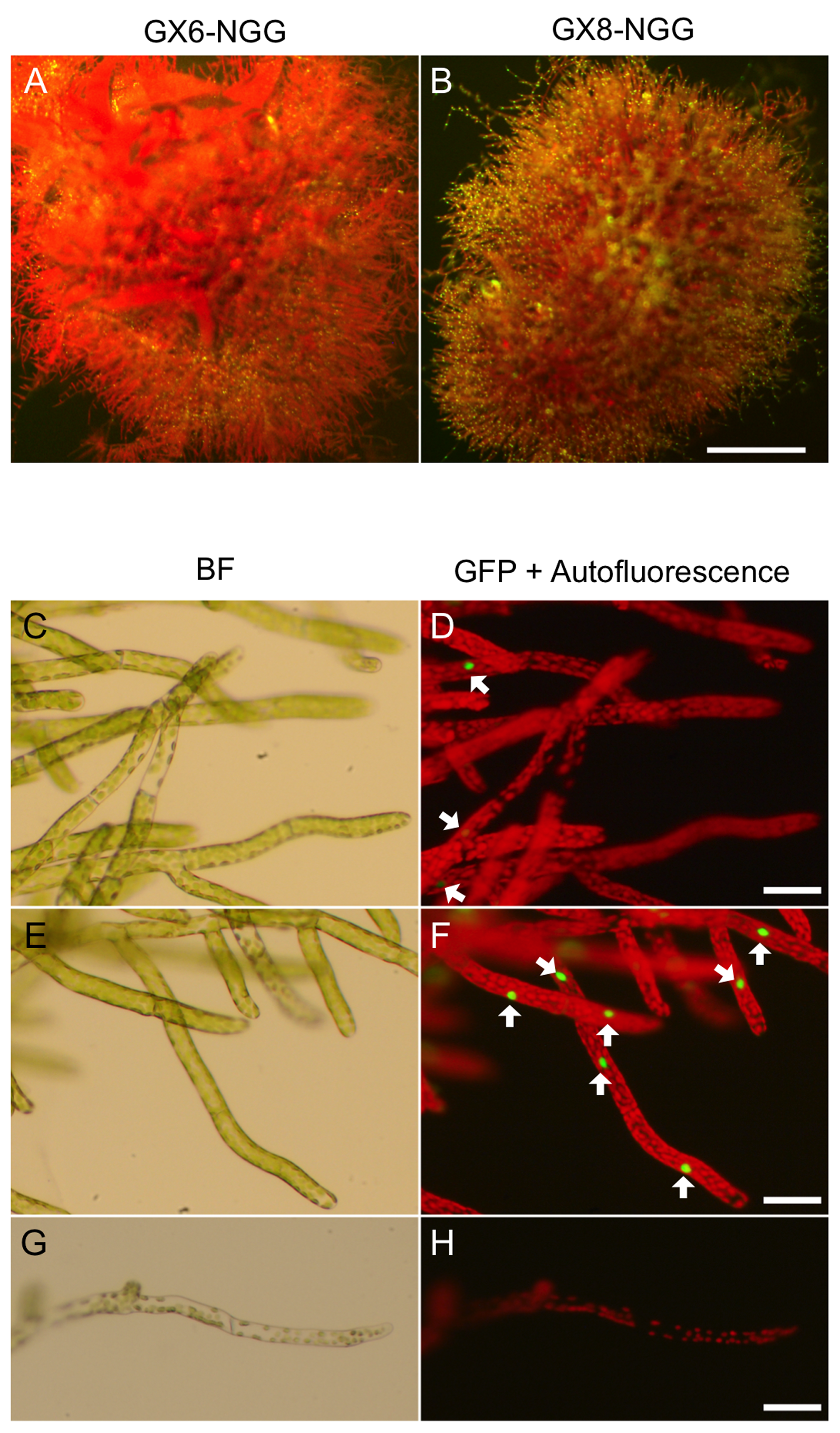

Supplement: Figure S3 — 8-day old protonemata of GX6-NGG and GX8-NGG lines and high-resolution images of chloronema and cauronema cells in GX6-NGG lines with β-estradiol. (A,B) Fluorescence images of 8-day old protonemata of GX6-NGG#63 (A) and GX8-NGG#4 (B) lines. Small amount of protonemata was inoculated on BCDAT agar under continuous light at 25°C for 7 days. And then, these were immersed in the water with 1 µM β-estradiol for 1 day before observation. Bright field (C, E, G) and fluorescence (D, F, H) images of chloronema (C, D, E, F) and cauronema (G, H) cells of GX6-NGG#63 lines. Arrows in D and F indicate nuclei with GFP signals. Bars: B = 1 mm. D, F, and H = 200 µm. (TIF) [file pone.0077356.s003.tif]

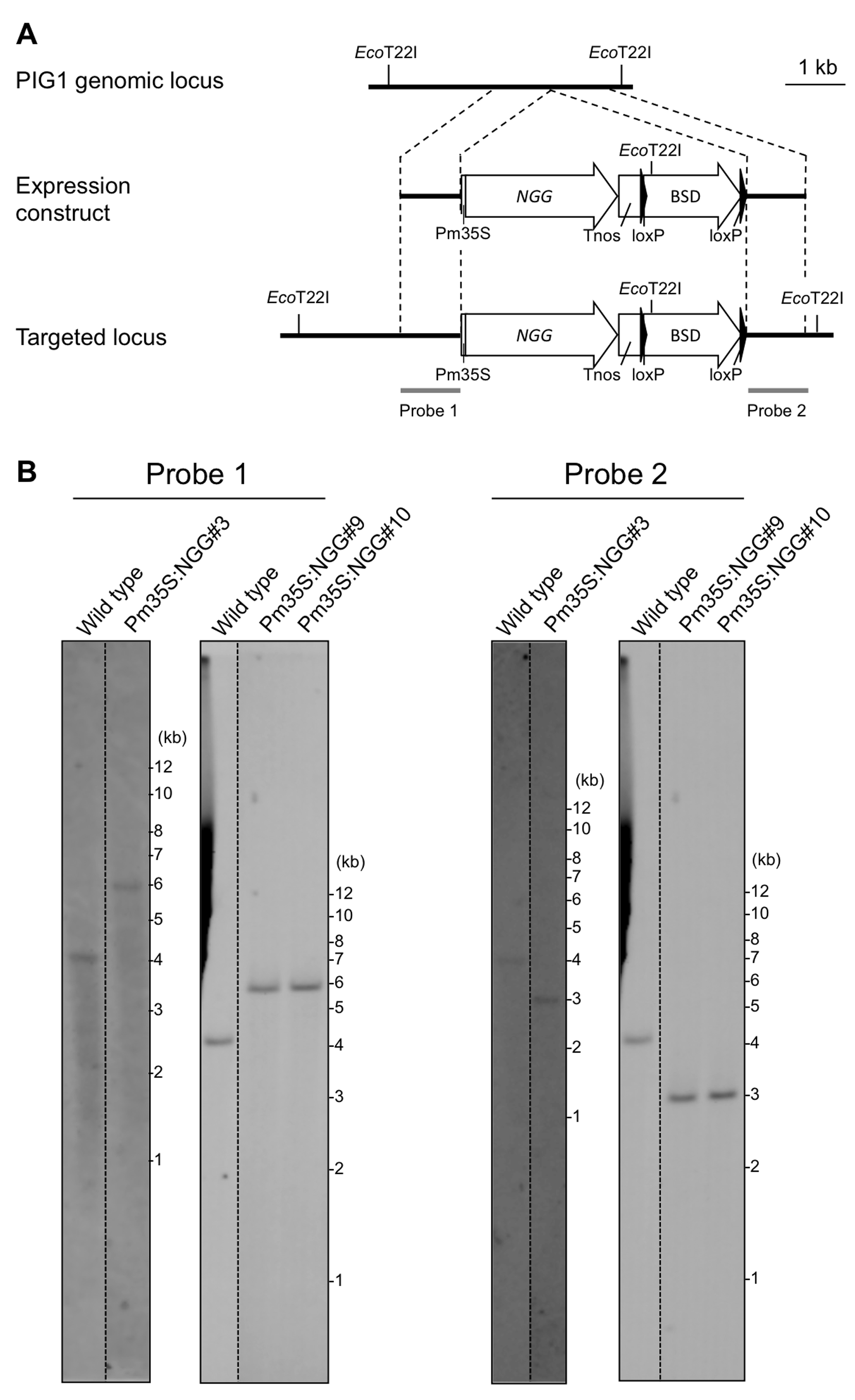

Supplement: Figure S4 — DNA gel blot analysis of transgenic P . patens Pm35S:NGG lines. (A) Schematic representation of the genomic locus and the construct. Pm35S: CaMV minimal 35S promoter [23], NGG: the NLS-GFP-GUS (NGG) fusion gene composed of a nuclear localization signal (NLS [39]:), the green fluorescent protein (sGFP [40]:) gene, and the uidA (GUS [41]:) gene, Tnos: a nos terminator [26], BSD: the blasticidin S deaminase gene cassette [26]. Gray bars indicate probe regions for DNA gel blot analyses. (B) DNA gel blot analyses of Pm35S:NGG transgenic P . patens lines. Each genomic DNA was digested with EcoT22I. (TIF) [file pone.0077356.s004.tif]

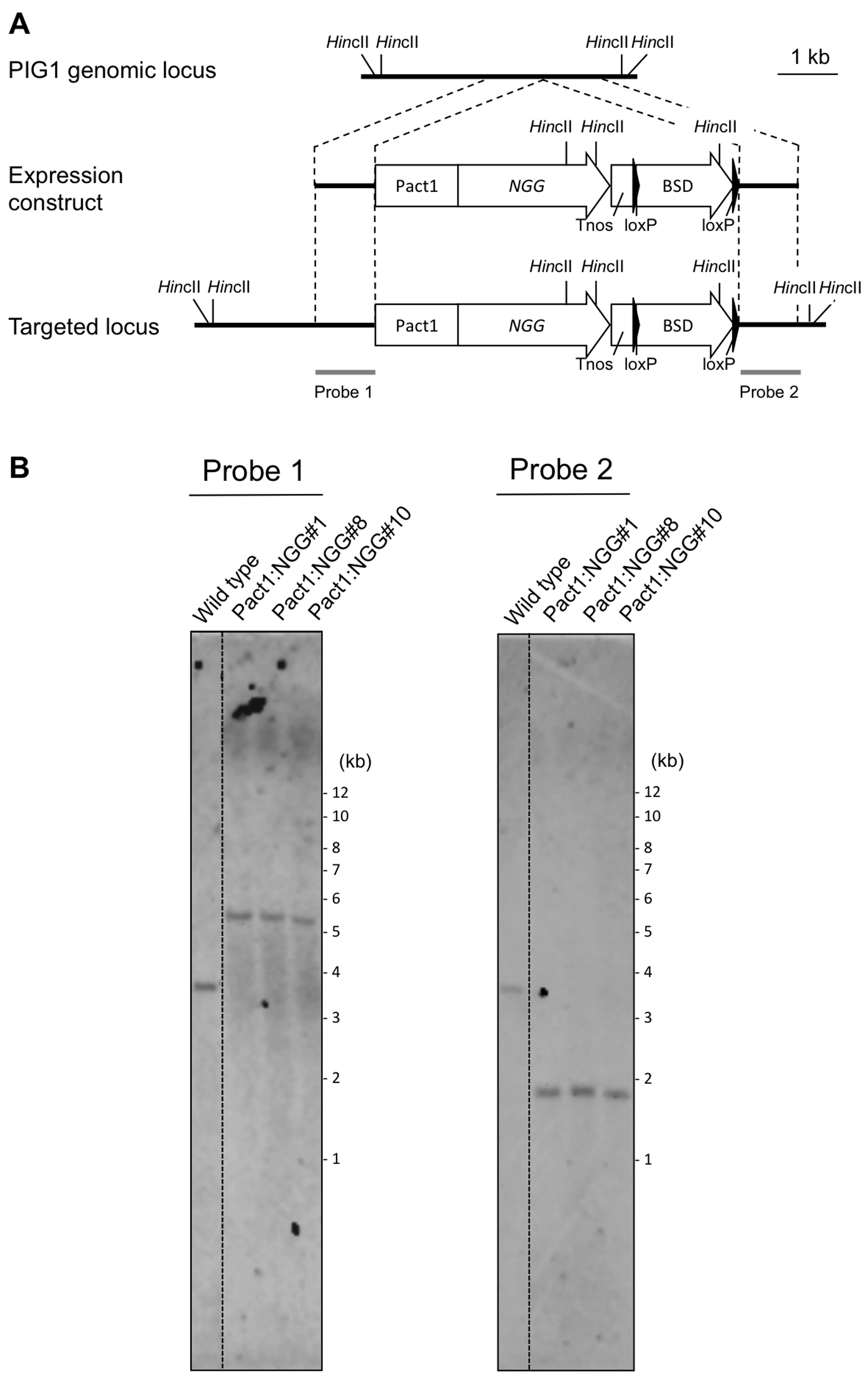

Supplement: Figure S5 — DNA gel blot analysis of transgenic P . patens Pact1:NGG lines. (A) Schematic representation of a genomic locus and the construct Pact1: the rice actin1 promoter [16,42], NGG: the NLS-GFP-GUS (NGG) fusion gene composed of a nuclear localization signal (NLS [39]:), the green fluorescent protein (sGFP [40]:) gene, and the uidA (GUS [41]:) gene, Tnos: a nos terminator [26], BSD: the blasticidin S deaminase gene cassette [26]. Other abbreviations are indicated in the legend of Figure 1. Gray bars indicate probe regions for DNA gel blot analyses. (B) DNA gel blot analyses of Pact1:NGG transgenic P . patens lines. Each genomic DNA was digested with HincII. (TIF) [file pone.0077356.s005.tif]

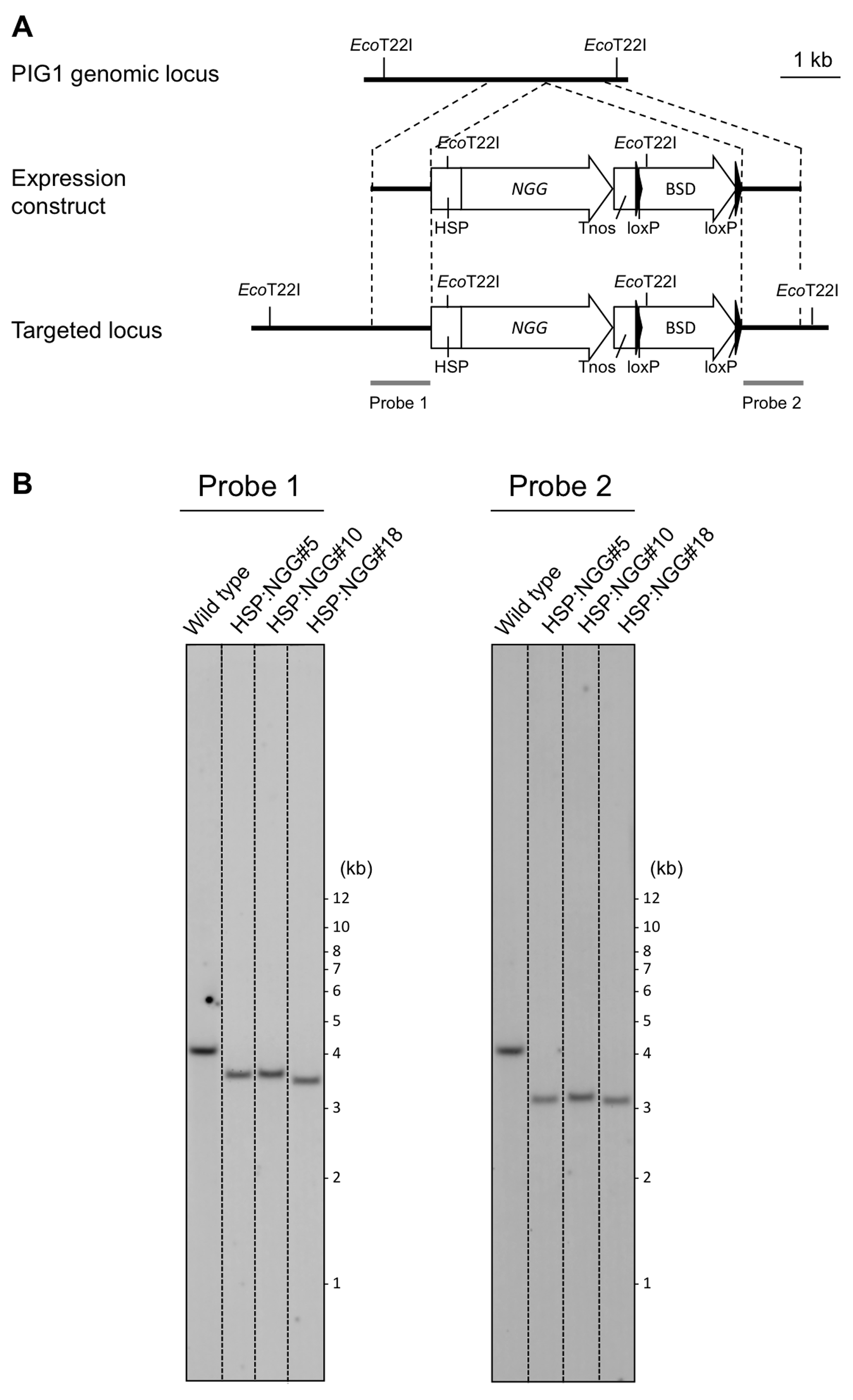

Supplement: Figure S6 — DNA gel blot analysis of transgenic P . patens HSP:NGG lines. (A) Schematic representation of a genomic locus and the construct. HSP: the soybean Gmhsp17.3B promoter [13,14], NGG: the NLS-GFP-GUS (NGG) fusion gene composed of a nuclear localization signal (NLS [39]:), the green fluorescent protein (sGFP [40]:) gene, and the uidA (GUS [41]:) gene, Tnos: a nos terminator [26], BSD: the blasticidin S deaminase gene cassette [26]. Gray bars indicate probe regions for DNA gel blot analyses. (B) DNA gel blot analyses of HSP:NGG transgenic P . patens lines. Each genomic DNA was digested with EcoT22I. (TIF) [file pone.0077356.s006.tif]

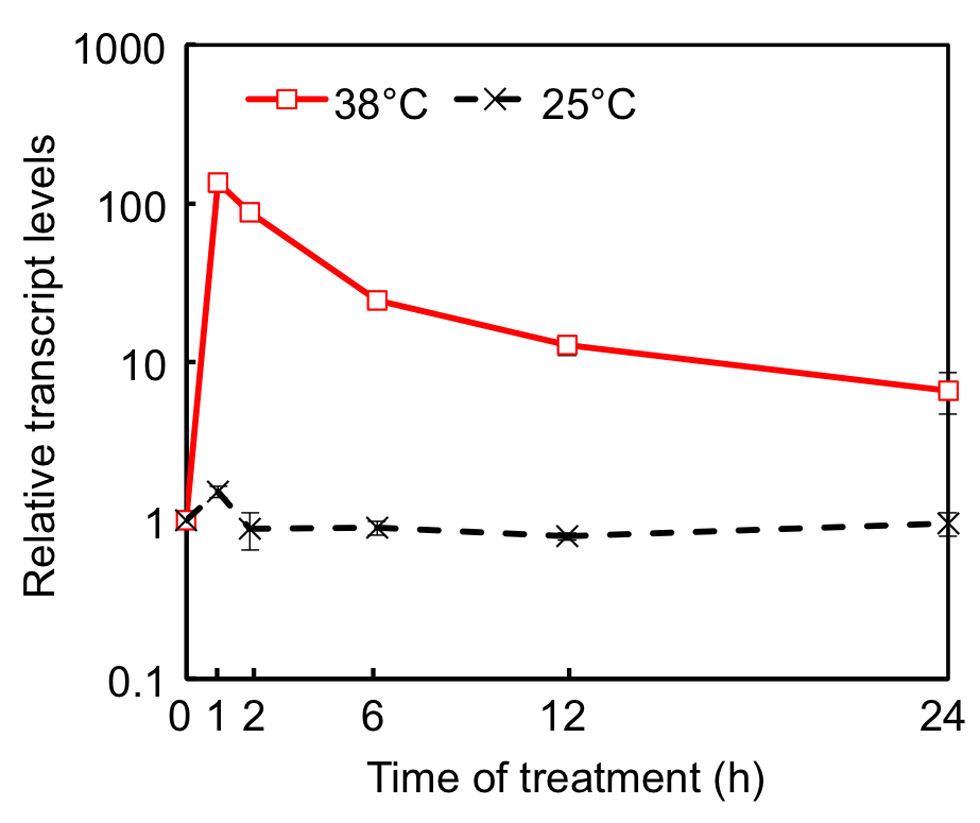

Supplement: Figure S7 — Time-course responsiveness to heat shock treatment of the HSP:NGG#5 line. Protonemata were incubated at 25°C (crosses) and 38°C (squares) for 0, 1, 2, 6, 12, and 24 h and then immediately collected. Relative transcript levels of NGG are normalized to TUA1 and then standardized to a normalized transcript level of protonemata at 0 h. Error bars indicate SD of the mean (n = 3). (TIF) [file pone.0077356.s007.tif]

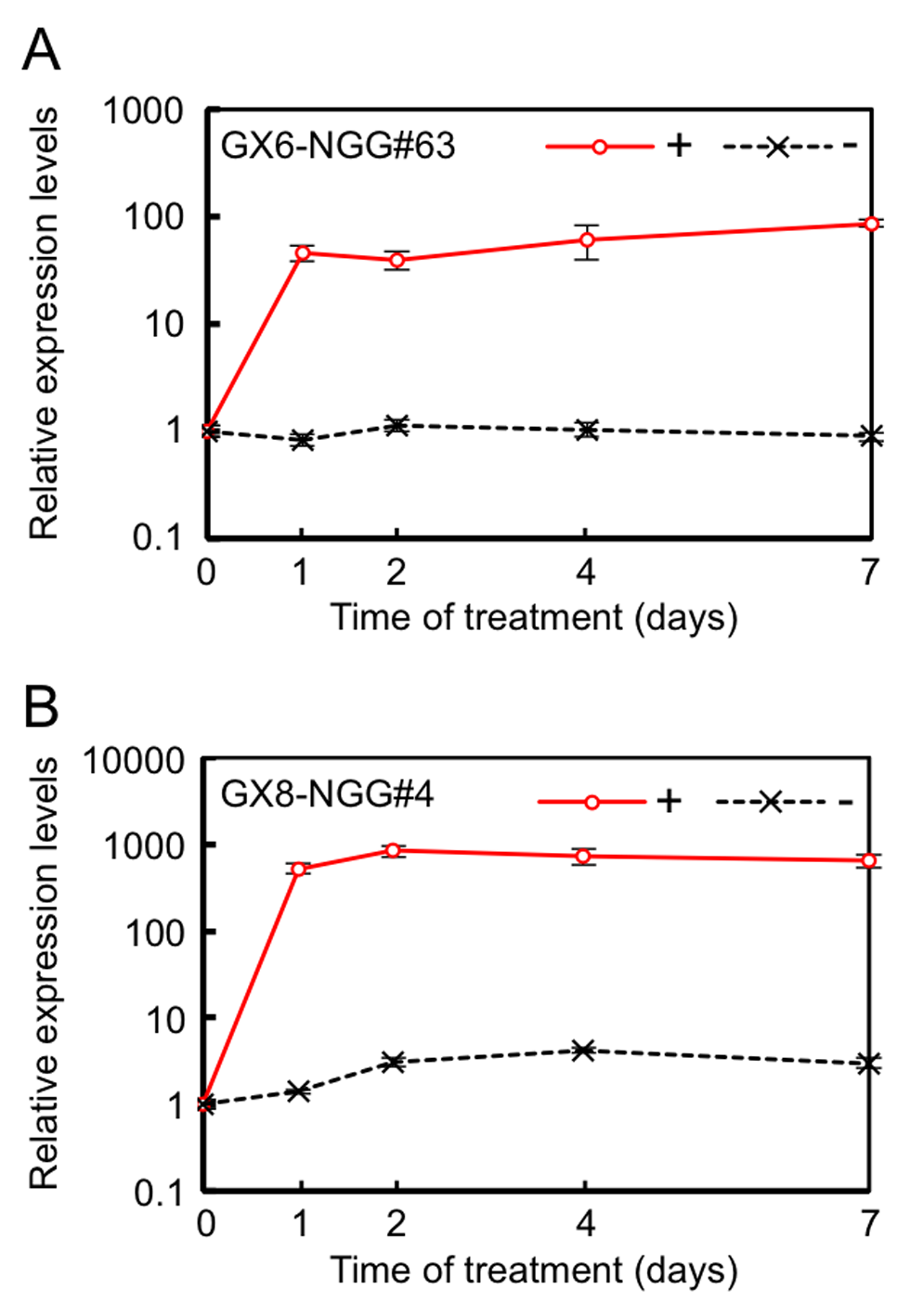

Supplement: Figure S8 — Time course responsiveness to β-estradiol in the GX6-NGG and GX8-NGG lines. (A, B) Relative transcript levels of NGG in protonemata of GX6-NGG#63 (A) and GX8-NGG#4 (B) lines. Relative transcript levels of NGG are normalized to TUA1 and then standardized to a normalized transcript level of protonemata at 0 h. Protonemata were immersed in water with (+: circles) or without (-: crosses) 1 µM β-estradiol and collected after 1, 2, 4, and 7 d. Error bars indicate SD of the mean (n = 3). (TIF) [file pone.0077356.s008.tif]

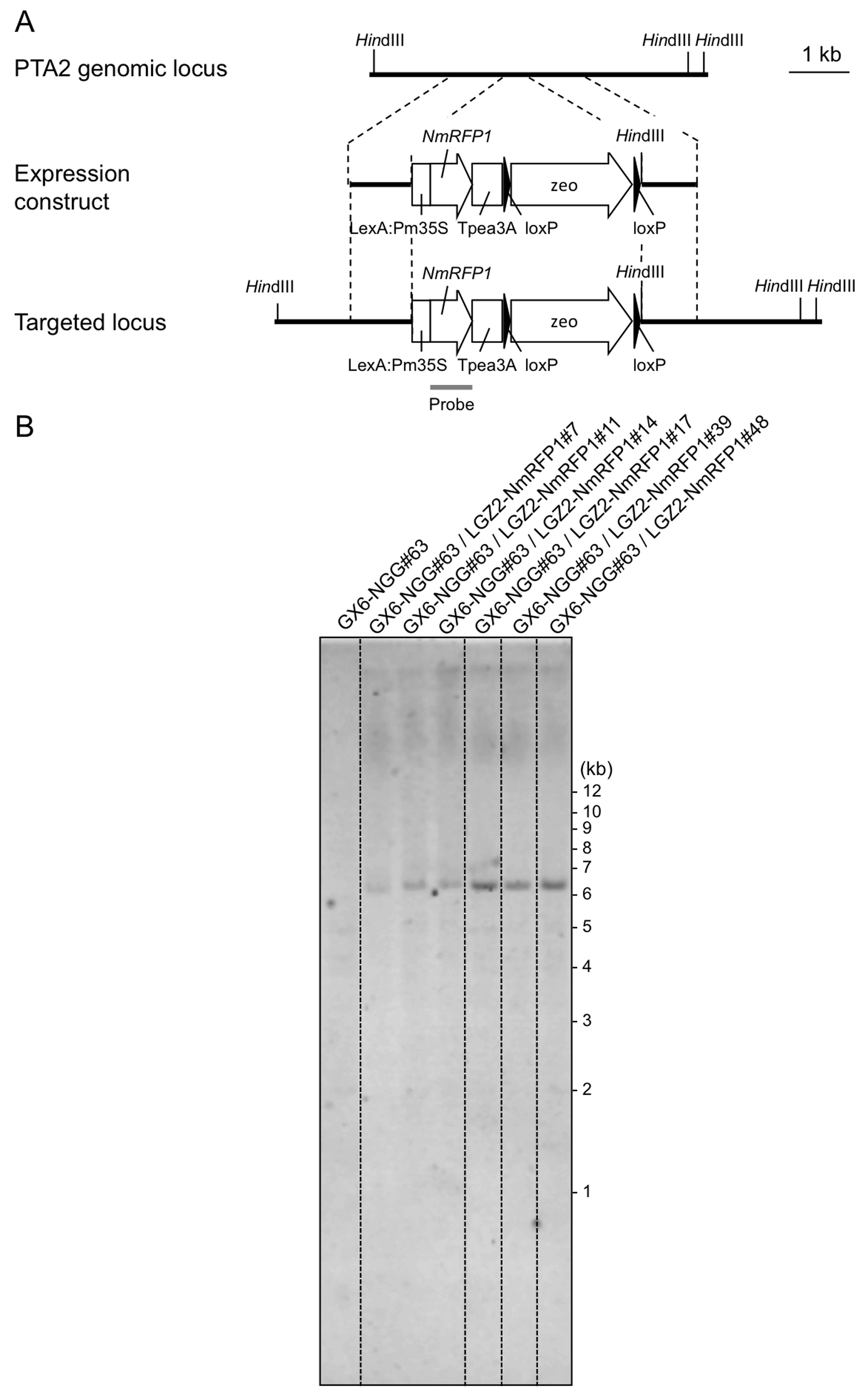

Supplement: Figure S9 — DNA gel blot analysis of transgenic P . patens GX6-NGG/LGZ2-NmRFP1 lines. (A) Schematic representation of a genomic locus and the construct. LexA:Pm35S: eight copies of LexA operators [22] connected to CaMV minimal 35S promoter [23], NmRFP1: the mRFP1 [43] gene with a nuclear localization signal [39], Tpea3A: a Tpea3A terminator [34], loxP: the sequences for site-specific recombination by Cre recombinase [55], zeo: the bleomycin resistant protein expression cassette [36]. A gray bar indicates a probe region for DNA gel blot analyses. (B) DNA gel blot analyses of GX6-NGG#63/LGZ2-NmRFP1 transgenic lines. Genomic DNA was digested with HindIII. (TIF) [file pone.0077356.s009.tif]
