# Supplementary material for: System for Stable β-Estradiol-Inducible Gene Expression in the Moss Physcomitrella patens
Source: PLoS One. 2013 Sep 27;8(9):e77356. doi: 10.1371/journal.pone.0077356 (PMC3785464; doi:10.1371/journal.pone.0077356)
Supplement: Table S1 — Primers for cloning of GX promoter regions. (PDF) [file pone.0077356.s010.pdf]

Table S1. Primers for cloning of GX promoter regions.

Promoter region		Sequence (5’ to 3’)
PGX6	Forward	TCATTGTTCTTCATTGTTTTCTATCA
	Reverse	TTCGCCTCCACTCGAAACTCCA
PGX8	Forward	TTTTGTTTGAGGAGGTTTTACTTTTTTG
	Reverse	CGCACCCGACGGCTCTGTTTTA
