# Supplementary material for: System for Stable β-Estradiol-Inducible Gene Expression in the Moss Physcomitrella patens
Source: PLoS One. 2013 Sep 27;8(9):e77356. doi: 10.1371/journal.pone.0077356 (PMC3785464; doi:10.1371/journal.pone.0077356)
Supplement: Table S2 — Primers for qPCR. (PDF) [file pone.0077356.s011.pdf]

Table S2. Primers for qPCR.

Target gene		Sequences (5' to 3')
NGG	Forward	GTCCGCCCTGAGCAAAGA
	Reverse	TCCAGCAGGACCATGTGATC
PpTUA1	Forward	CGTAGGAGGGACCAGTTTGG
	Reverse	TGCATTCATCCCCGAGTCA
NmRFP1	Forward	CGAGGTCAAGACCACCTACATG
	Reverse	TGGGAGGTGATGTCCAGCTT
